# Pan-Fibrotic Gene Expression Signature in Major Chronic Diseases by Integrative Bulk and Single-Cell Transcriptomic Analyses

**DOI:** 10.3390/ijms27198755

**Published:** 2026-09-30

**Authors:** Mayra Alejandra Jaimes-Campos, Rafael Stroggilos, Joost P. Schanstra, Antonia Vlahou, Manon Brunet, Jean-Christophe Jonas, Mohammed Bensellam, Harald Mischak, Agnieszka Latosinska

**Affiliations:** 1 Department of Biomarker Research, Mosaiques Diagnostics GmbH, D-30659 Hannover, Germany; jaimes@mosaiques-diagnostics.com (M.A.J.-C.);; 2 Systems Biology Center, Biomedical Research Foundation, Academy of Athens, 11527 Athens, Greece; 3 Institut National de la Santé et de la Recherche Médicale (INSERM), Institute of Metabolic and Cardiovascular Diseases, 31432 Toulouse, France; 4 Faculty of Health, Université Toulouse III Paul-Sabatier, 31432 Toulouse, France; 5 Pôle d’Endocrinologie, Diabète et Nutrition, Institut de Recherche Expérimentale et Clinique, Université Catholique de Louvain, B-1200 Brussels, Belgium

**Keywords:** chronic diseases, fibrosis, transcriptomics, single cell, biomarkers

## Abstract

Fibrosis is a shared feature of chronic liver, kidney and heart disease, yet no molecular signature captures the mechanisms common to these organs. We integrated human microarray, bulk and single-nucleus RNA sequencing (snRNA-seq) data across 2022 unique tissue samples to identify a conserved pan-fibrotic signature. The pan-fibrotic signature was defined in a microarray discovery cohort (*n* = 1051) and verified in a microarray liver (*n* = 325), two RNA-seq (*n* = 414) and four integrated snRNA-seq validation datasets (*n* = 69). Diagnostic models were built using five machine learning approaches in separate RNA-seq cohorts (training *n* = 209; liver validation *n* = 70; kidney validation *n* = 24). 492 fibrosis-associated genes were identified across organs, including 23 hallmark genes, a subset of which correlated with disease severity. A novel finding was consistent metallothionein (*MT1E*, *MT1X*) downregulation; snRNA-seq resolved cell-type-specific expression of 12 hallmark genes, including *MT1E*/*MT1X* predominantly expressed in hepatocytes and liver sinusoidal endothelial cells. Elastic Net performed best across all three gene sets (AUROC 0.765–0.830), with scores correlating significantly with interstitial fibrosis and tubular atrophy (%IFTA; rho 0.58–0.73). This study defines a cross-organ pan-fibrotic signature, providing potential candidate therapeutic targets and biomarkers applicable across tissues affected by fibrosis.

## 1. Introduction

Fibrosis is a pathological process characterised by disrupted extracellular matrix (ECM) homeostasis, leading to excessive accumulation of ECM components. The progression of fibrosis is a highly dynamic and multi-factorial process. It represents the final common pathway in a wide range of chronic diseases affecting vital organs, including the liver, kidney, lung, and heart. Progressive fibrosis results in tissue remodelling, organ dysfunction, and ultimately organ failure, representing a substantial societal and economic burden worldwide [1].

Despite significant unmet needs, the development of effective anti-fibrotic therapies remains limited by the lack of early diagnostic and prognostic biomarkers, as well as a limited number of validated molecular targets [2]. Furthermore, understanding the complex molecular mechanisms underlying fibrosis initiation and progression is essential for developing new treatment strategies [3,4,5]. While tissue-specific mechanisms have been extensively studied, the processes that sustain chronic fibrosis, believed to follow conserved molecular principles across organs, remain poorly understood [2]. Therefore, identifying common molecular signatures and core signalling pathways in humans with cross-tissue relevance, continues to be a research priority.

Advances in next-generation sequencing and transcriptomic profiling have led to the characterisation of numerous fibrosis-associated genes (e.g., *TGF-β1*, *COL1A1*, *LOXL2*, *miR-21*, among others), uncovering critical drivers and key mechanisms [3,6,7,8]. Furthermore, several signalling pathways have been implicated in the development and progression of fibrosis, and some have been described in multiple tissues and disease contexts, including among others, ECM remodelling, TGFβ–SMAD, Hedgehog, Notch, and Wnt/β-catenin signalling, as well as fibrogenic mediator connective tissue growth factor (CTGF/CCN-2) [2,9,10]. In contrast, regulatory pathways, including phosphoinositol-3 kinase (PI3K)/Akt, Hippo, peroxisome proliferator-activated receptors (PPARs), and farnesoid X receptor (FXR) [9,11,12], exhibit substantial variability across tissues. Despite the growing number of tissue-specific fibrosis markers and pathways, to the best of our knowledge, there are no established gene signatures or pathways that consistently capture fibrosis across multiple human organs. Identifying and validating such gene signatures or pathways and understanding how they integrate in the development and progression of fibrosis are essential for uncovering the underlying biology and guiding the development of effective, broadly applicable anti-fibrotic therapies.

In this study, we performed a comprehensive, cross-platform multi-layer transcriptomic analysis of human fibrosis across multiple organs, including the liver, kidney and heart, by integrating microarray, bulk RNA sequencing (RNA-seq), and single-nucleus RNA-seq (snRNA-seq) datasets. By employing multi-centre cohort and cross-platform data from a total of 2022 unique samples, and exploring different levels including single gene, pathway and network, we identified and verified a novel multi-organ fibrosis-associated gene signature (hallmark genes) that reflects conserved transcriptional alterations across organs. To our knowledge, this is the first study to identify and validate a pan-fibrotic signature conserved across the liver, kidney, and heart in humans using transcriptomics data. Notably, our results suggested that fibrosis may be driven not only by excessive collagen production, but also by impaired ECM degradation. Our findings support this dual mechanism, highlighting the altered expression of genes involved in formation of collagen fibrils such as *COL5A1*, *AEBP1*, *SOX4*, *THBS2*, *MFAP4*, *PLA2G4C*, *AZGP1*, alongside the downregulation of metallothioneins (MT; *MT1X* and *MT1E*), which have been associated with reduced MMP9 activation. Subsequently, to assess the translational potential of the identified signatures, we further constructed diagnostic models discriminating between fibrosis and no fibrosis, using five ML algorithms, demonstrating promising performance in independent validation datasets, with model scores significantly correlating with the %IFTA. Collectively, this study provides additional insights into molecular pathophysiology that may enable the discovery of conserved biomarkers applicable across multiple organ systems, support early diagnosis, and guide the design and development of broadly effective anti-fibrotic therapies.

## 2. Results

### 2.1. Pan-Fibrotic Gene-Expression Signature Identification

**Figure 1:**
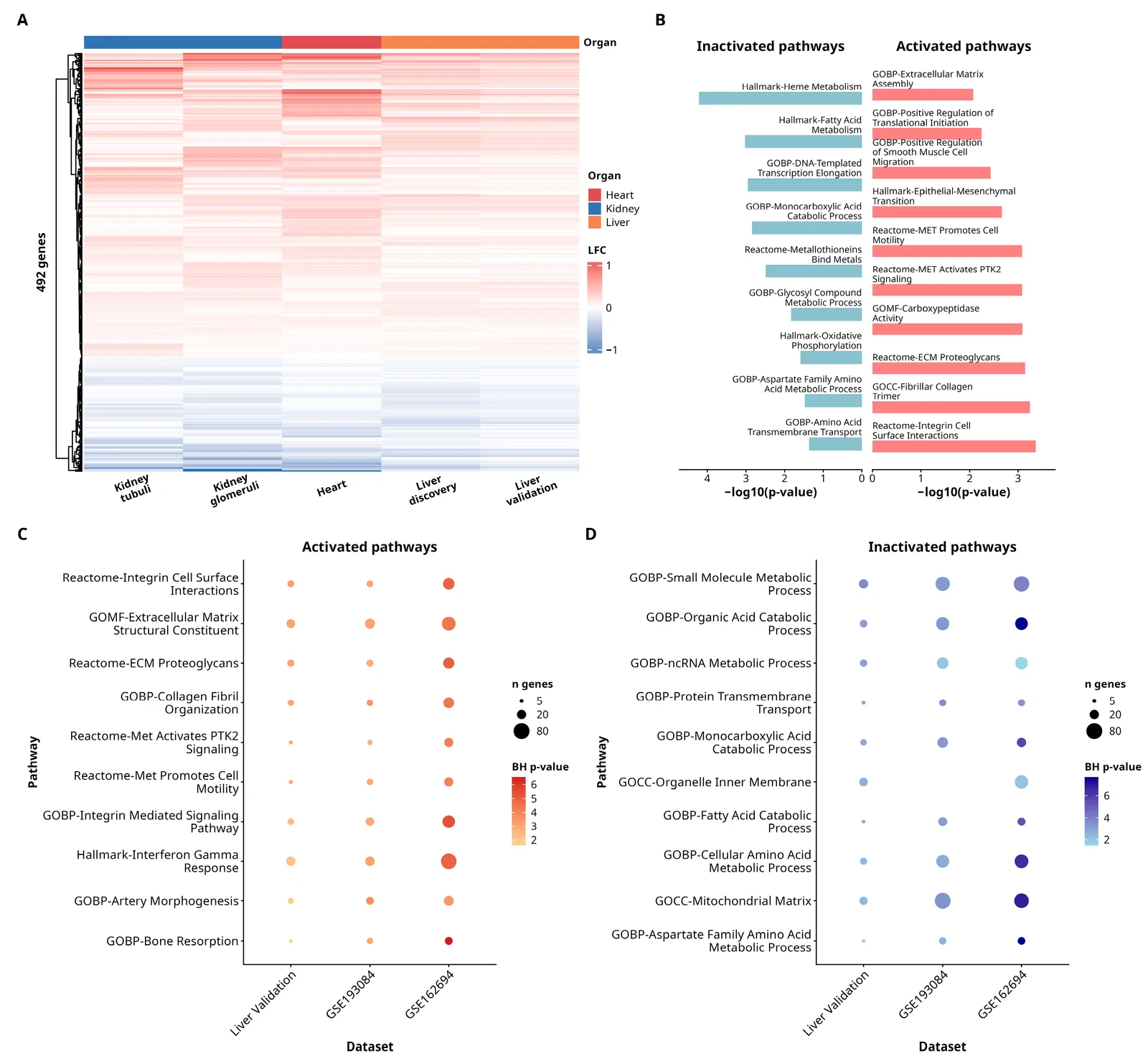
Identification and validation of a pan-fibrotic signature/pathways using multi-centric bulk microarray and RNA-seq datasets. Heatmap representing the (**A**) 492 fibrotic signatures identified in the discovery and validation cohorts. Z-scaled fold changes are shown, with increased expression in fibrotic tissues depicted in red and decreased expression in blue. (**B**) Bar plot summarising the top activated (red) and inhibited (blue) pathways across the discovery and initial validation cohorts, based on pathway enrichment analysis. (**C**) Dot plot showing the top activated pathways and (**D**) top inhibited pathways validated across the liver RNA-seq datasets (GSE193084 and GSE162694). Dot size represents the number of significant genes contributing to each pathway, while colour indicates statistical significance (−log10(BH *p*-value)). The top enriched pathways were selected based on the lowest adjusted *p*-value in the microarray initial validation cohort and the highest coverage (defined as the proportion of genes contributing to each pathway). When multiple pathways were associated with the same biological process, the one with the highest coverage was retained. Abbreviations: LFC, log2 fold change; GOBP, Gene Ontology Biological Process; GOCC, Gene Ontology Cellular Component; GOMF, Gene Ontology Molecular Function; Hallmark, MSigDB Hallmark gene set collection; Reactome, Reactome pathway database; ECM, extracellular matrix; MET, MET proto-oncogene receptor tyrosine kinase; PTK2, protein tyrosine kinase 2 (focal adhesion kinase); ncRNA, non-coding RNA; BH, Benjamini–Hochberg; GSE, Gene Expression Omnibus series accession.

To determine a pan-fibrotic gene signature, we re-analysed multi-centric bulk microarray datasets of human tissue samples from fibrotic and non-fibrotic conditions. Organ-specific analyses enabled the identification of fibrotic gene expression signatures for each organ. These signatures comprised 4523 differentially expressed genes in the heart, 1769 in kidney tubules, 2391 in kidney glomeruli, and 1777 in the liver (Table S1). We subsequently defined cross-organ features as genes that reached statistical significance (BH *p* < 0.05) in at least one organ-specific signature and additionally showed the same direction of change across all remaining organs, identifying 572 features (Table S2). To further assess the robustness of these findings, four additional bulk microarray datasets of liver tissues with known degrees of fibrosis were analysed and confirmed the concordant regulation of 492 of 572 genes, thereby establishing a pan-fibrotic gene expression signature (Figure 1A; Table S3).

To investigate the molecular mechanisms associated with fibrosis, we performed pathway activation analysis using the expression profiles of the 492 genes in fibrotic versus non-fibrotic conditions across each organ. The analysis identified 172 activated and 143 inhibited pathways that were significantly enriched across all organs in both the discovery and initial validation cohorts, with consistent activation trends (Table S4). The most significant activated pathways were related to ECM remodelling, including collagen fibril organisation, ECM proteoglycans, and positive regulation of smooth muscle cell migration, highlighting the central role of ECM-related structural remodelling in fibrosis (Figure 1B). In contrast, inhibited pathways were primarily associated with metabolic processes. Heme metabolism, fatty acid metabolism, and oxidative phosphorylation were consistently inhibited in fibrotic tissues. Notably, we also observed attenuation of metallothioneins bind metals, zinc ion homeostasis pathways and cellular response to zinc ion, along with transcriptional regulation pathways, including DNA-templated transcription elongation (Figure 1B; Table S4). These findings suggest a conserved pattern of ECM activation accompanied by metabolic dysregulation and impaired zinc ion signalling, which are consistently associated with fibrosis across organs. Notably, the consistent attenuation of zinc ion homeostasis pathways in this pan-fibrotic gene expression signature further implicates an unexpected role for MT family members and zinc transporters in fibrosis.

### 2.2. Pathway-Level Validation Across Datasets

We next assessed whether these fibrosis-associated pathways were conserved in the two independent RNA-seq datasets. Among the pathways that showed a consistent activation or inhibition status across all microarray comparisons, 74 activated and 13 inhibited pathways were confirmed in the RNA-seq datasets (BH *p* < 0.05 and same trend in the fold change), representing the most conserved molecular processes associated with fibrosis (Table S5). Consistent with the microarray findings, the most significantly activated pathways were associated with ECM remodelling and immune response (Figure 1C; Table S5). The validated inhibited pathways were primarily dominated by metabolic, mitochondrial, as well as transcription-related pathways, supporting a conserved pattern of metabolic disruption of cellular metabolism, which may contribute to impaired cellular function in fibrotic tissues (Figure 1D; Table S5).

### 2.3. Hallmark Genes

**Figure 2:**
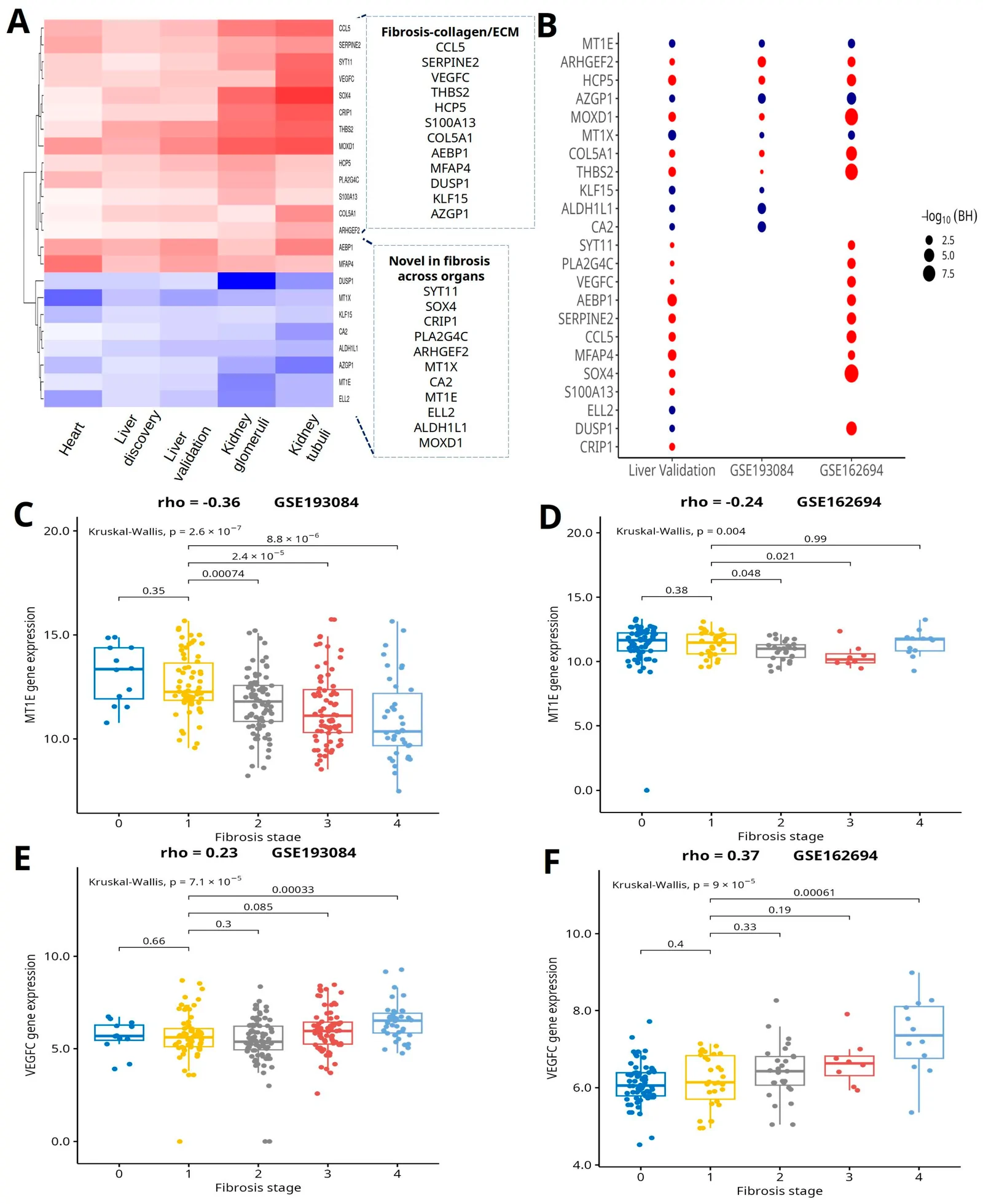
Hallmark elements of the pan-fibrotic gene-expression signature. (**A**) 23 hallmark molecular features within the pan-fibrotic gene expression signature, with genes showing increased expression in fibrosis represented in red and those with decreased expression in blue (**B**) Validation of the 23 fibrotic-associated genes in two independent RNA-seq liver datasets (GSE193084 and GSE162694). Each dot represents a significant gene (BH *p* < 0.05), with genes showing increased expression in fibrosis represented in red and those with decreased expression in blue. (**C**) Box plots illustrating the association of *MT1E* expression with fibrosis progression in the GSE193084, and (**D**) in the GSE162694 RNA-seq liver validation datasets. (**E**) Association of *VEGFC* expression with fibrosis progression in the GSE193084, and (**F**) in the GSE162694 RNA-seq liver validation datasets. Rho values indicate Spearman correlation coefficients, and *p*-values were calculated using the Kruskal–Wallis test. Abbreviations: ECM, extracellular matrix; BH, Benjamini–Hochberg; GSE, Gene Expression Omnibus series accession; rho, Spearman’s rank correlation coefficient.

To identify hallmark molecular features within the pan-fibrotic gene expression signature (492 genes), we focused on genes that were significantly and consistently differentially expressed (BH *p* < 0.05) across all comparisons in both the discovery and initial validation sets. This approach identified 23 genes that constitute the core of the pan-fibrotic gene expression signature (Figure 2A; Tables S3 and S6). Several of these genes, such as *CCL5*, *SERPINE2*, *THBS2*, *COL5A1*, *VEGFC*, are well-established fibrosis-associated genes involved in ECM remodelling, supporting the validity of the approach. Notably, the analysis also identified novel genes not previously implicated in fibrosis across the heart, liver and kidney, including *MOXD1*, *SOX4*, *SYT11*, *CRIP1*, *PLA2G4C*, *ARHGEF2*, *CA2*, and *ELL2*, and members of the MT1 family (*MT1X* and *MT1E*; Figure 2A; Table S6). The identification of *MT1E* and *MT1X* within this conserved core cross-organ signature highlights a previously unrecognised association between MTs and human fibrosis. Because the discovery and initial validation microarray cohorts share the same 140 control samples, we assessed whether the reproducibility of the hallmark genes depended on this overlap. Liver differential expression analyses were recomputed on the ComBat matrix in the two cohorts with no overlapping controls, comparing the results with those of the primary analysis. Under the new analysis, 531 features met the cross-organ criterion. Of these, 485 overlapped with the primary analysis (84.8%), along with 46 newly qualifying genes; 378 showed concordant regulation in the validation cohort, retaining 367 genes; and 12 reached BH *p* < 0.05 across all five comparisons (Table S2). Eleven of the 23 published hallmark genes overlapped with the primary analysis (*MFAP4*, *MT1X*, *ELL2*, *MOXD1*, *CCL5*, *SYT11*, *AEBP1*, *HCP5*, *S100A13*, *THBS2* and *CA2*), and *BGN*, a biglycan proteoglycan central to extracellular matrix assembly, additionally met all criteria. Of the 12 hallmark genes not retained, 11 fail on the liver discovery comparison alone and only one (*VEGFC*) on the liver validation comparison (Table S3). Critically, the effect sizes are essentially unchanged. Across the pan-fibrotic signature, log2 fold changes from the stratified analyses correlate closely with those of the main analyses (Pearson r = 0.958 for discovery and r = 0.970 for validation), rising to r = 0.987 and r = 0.992 among the 23 hallmark genes. Therefore, the genes that no longer reach significance showed the same magnitude and direction of change as the primary analysis, and their loss reflects the smaller number of samples in the control groups rather than any inflation of the original result by the shared samples. Consequently, the subsequent analyses were conducted using the 140 controls that were common to both cohorts.

We further evaluated the 23 genes identified in the primary analysis in a second, cross-platform validation cohort comprising two independent RNA-seq datasets with fibrosis stage annotations. Nineteen genes were validated in at least one of the two RNA-seq datasets, showing significant differential expression (BH *p* < 0.05) with consistent fold change direction as in the microarray analysis (Figure 2B; Table S6). Six genes (*MT1E*, *AZGP1*, *MT1X*, *KLF15*, *ALDH1L1*, and *CA2*) were consistently downregulated in fibrosis, while thirteen (*ARHGEF2*, *HCP5*, *MOXD1*, *COL5A1*, *THBS2*, *SYT11*, *PLA2G4C*, *VEGFC*, *AEBP1*, *SERPINE2*, *CCL5*, *MFAP4*, and *SOX4*) were upregulated. The concordant downregulation of *MT1E* and *MT1X* across datasets is particularly notable and, to the best of our knowledge, has not been previously reported in human fibrotic disease.

We further investigated the relationship between the gene expression levels and fibrosis stage in both RNA-seq datasets. Fifteen of the nineteen validated genes showed a significant correlation with fibrosis severity (BH *p* < 0.05). Expression of *ARHGEF2*, *HCP5*, *MOXD1*, *COL5A1*, *THBS2*, *SYT11*, *PLA2G4C*, *VEGFC*, *AEBP1*, *CCL5*, *MFAP4*, and *SOX4* was positively correlated with fibrosis, whereas *MT1E*, *AZGP1*, and *MT1X* were negatively correlated. These consistent patterns across datasets further support the potential of these genes as conserved, cross-organ biomarkers and as candidates for further investigation in fibrotic progression (Figure 2C–F and Figure S1A–H; Table S7). Collectively, these results support the biological relevance of the pan-fibrotic gene expression signature and indicate that it captures core mechanisms associated with fibrotic progression across tissues.

### 2.4. Cell-Type Resolution

**Figure 3:**
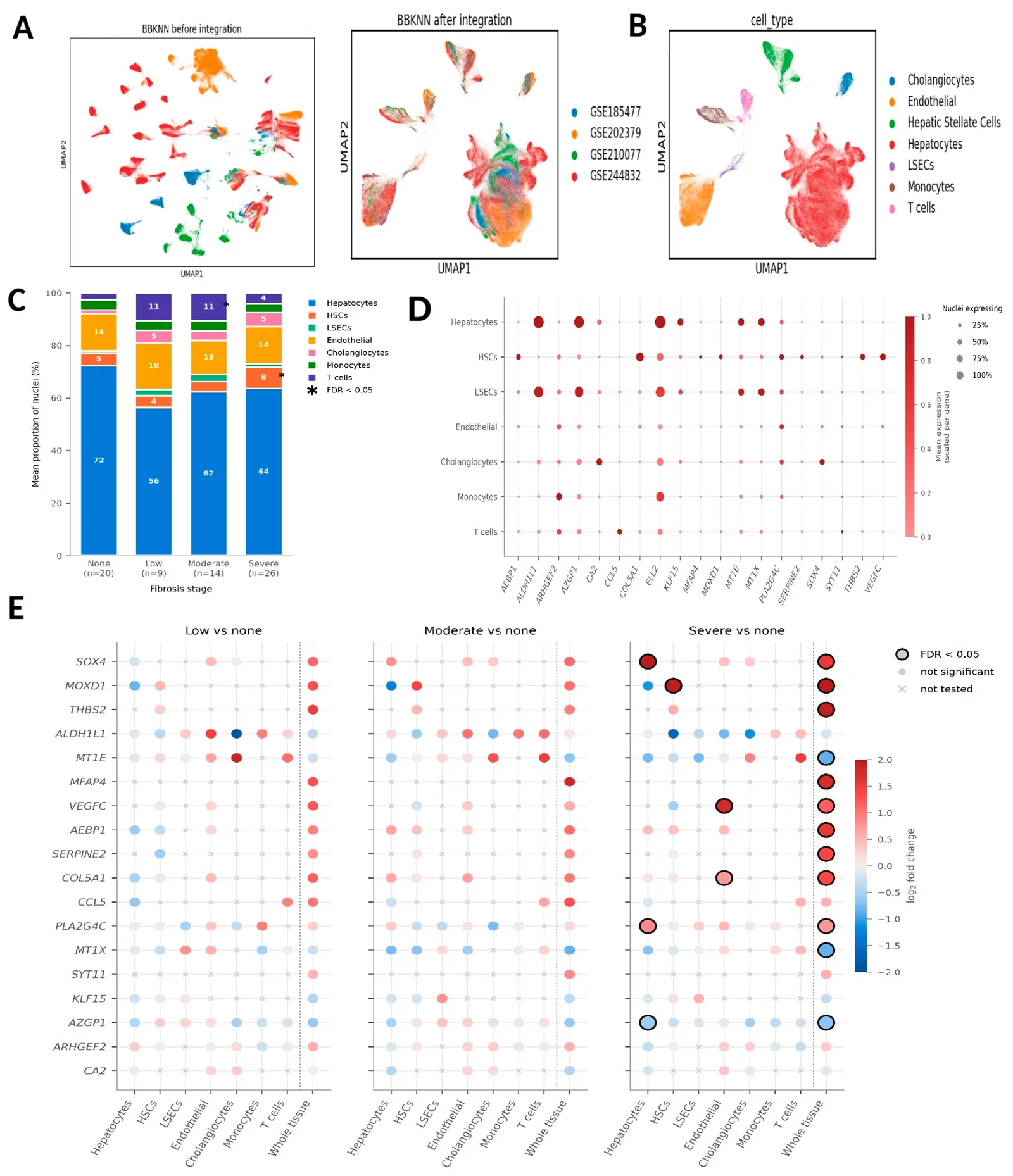
Single-cell validation of the pan-fibrotic gene expression signature in an integrated multi-cohort human liver snRNA-seq dataset. (**A**) UMAP plots showing the unintegrated (**left** panel) and integrated (**right** panel) snRNA-seq datasets from four independent cohorts (total cells = 249,088). (**B**) UMAP displaying the annotated liver cell types identified following integration. (**C**) Proportional distribution of liver cell types across fibrosis stages in the integrated multi-cohort snRNA-seq dataset. Proportions were computed per patient and compared using limma with dataset as a covariate; asterisks denote FDR < 0.05. (**D**) Dot plot showing cell-type-specific expression patterns of the 19 validated hallmark pan-fibrotic genes. Dot size indicates the percentage of nuclei expressing each gene and colour indicates mean expression, scaled per gene. (**E**) Patient-level pseudobulk differential expression for each fibrosis stage relative to no fibrosis, by cell type and in whole tissue. Colour indicates the log_2_ fold change; black rings mark comparisons with FDR < 0.05, small grey dots mark non-significant comparisons, and crosses mark comparisons that were not tested (fewer than five patients per group after the ≥10-nuclei filter). Abbreviations: snRNA-seq, single-nucleus RNA sequencing; BBKNN, batch balanced k-nearest neighbours; UMAP, Uniform Manifold Approximation and Projection; HSCs, hepatic stellate cells; LSECs, liver sinusoidal endothelial cells; FDR, false discovery rate; GSE, Gene Expression Omnibus series accession.

To achieve cell-level resolution, we integrated four human snRNA-seq liver dataset and investigated cell-type-specific expression patterns of the hallmark genes (Figure 3A). Major hepatic cell populations were identified and annotated using canonical markers, including hepatocytes, hepatic stellate cells (HSCs), liver sinusoidal endothelial cells (LSECs), endothelial cells, cholangiocytes, monocytes and T cells, supporting the reliability of the snRNA-seq datasets used (Figure 3B). Moreover, to enable a direct comparison with bulk RNA-seq, we generated sample-level pseudo-bulk profiles from the snRNA-seq data by aggregating counts from all nuclei across cell types belonging to the same patient. Canonical fibrotic markers (e.g., *COL6A2*, *COL5A1*, *COL3A1*, *COL1A1* and *ACTA1*), along with the hallmark genes, showed concordant upregulation in fibrotic versus non-fibrotic samples in both bulk RNA-seq and pseudo-bulk snRNA-seq, with similar magnitudes of change. These findings indicate that the global fibrotic transcriptional signal is preserved in the snRNA-seq data, which suggests that there has been no substantial loss of specific cell types during the single-nucleus preparation process.

Cell type composition was compared across fibrosis severity stages using per-patient proportions, with dataset included as a covariate. HSCs increased from 4.9% of nuclei in no fibrosis to 8.2% in severe fibrosis (BH *p* = 4.3 × 10^−4^), and T cells from 2.5% to 10.6% in moderate fibrosis (BH *p* = 0.045), consistent with stellate cell expansion and lymphocyte infiltration in fibrotic liver (Figure 3C). No significant change was observed for hepatocytes, LSECs, endothelial cells, cholangiocytes or monocytes. All major hepatic cell populations contributed to the expression of the hallmark genes, with the exception of *HCP5*, which was not identified in the snRNA-seq datasets (Figure 3D).

Further differential expression analysis, performed at the patient level, identified twelve out of the nineteen genes shortlisted from the bulk RNA analysis as significantly changed also in the fibrotic snRNA-seq dataset, with consistent fold change directionality between fibrotic and non-fibrotic conditions (Figure 3E). The shortlisted genes included *MOXD1*, *THBS2*, *COL5A1*, *AEBP1*, *SERPINE2*, *SOX4*, *AZGP1*, *MFAP4*, *VEGFC*, *PLA2G4C*, *MT1E* and *MT1X* (Table S8; Figure 3E). Cell-type-resolved analysis identified increased expression of *SOX4* and *PLA2G4C*, and reduced expression of *AZGP1* in hepatocytes, suggesting cell-type-specific regulatory roles in fibrotic progression. Furthermore, an increase in *VEGFC* and *COL5A1* was observed in endothelial cells, while *MOXD1* demonstrated an increase in HSCs in severe fibrosis (Figure 3E). MT family members, involved in zinc ion homeostasis (e.g., *MT1X*, *MT1E*), were predominantly expressed in hepatocytes and LSECs, and were significantly reduced at the tissue level (Figure 3D).

### 2.5. Network Connectivity

**Figure 4:**
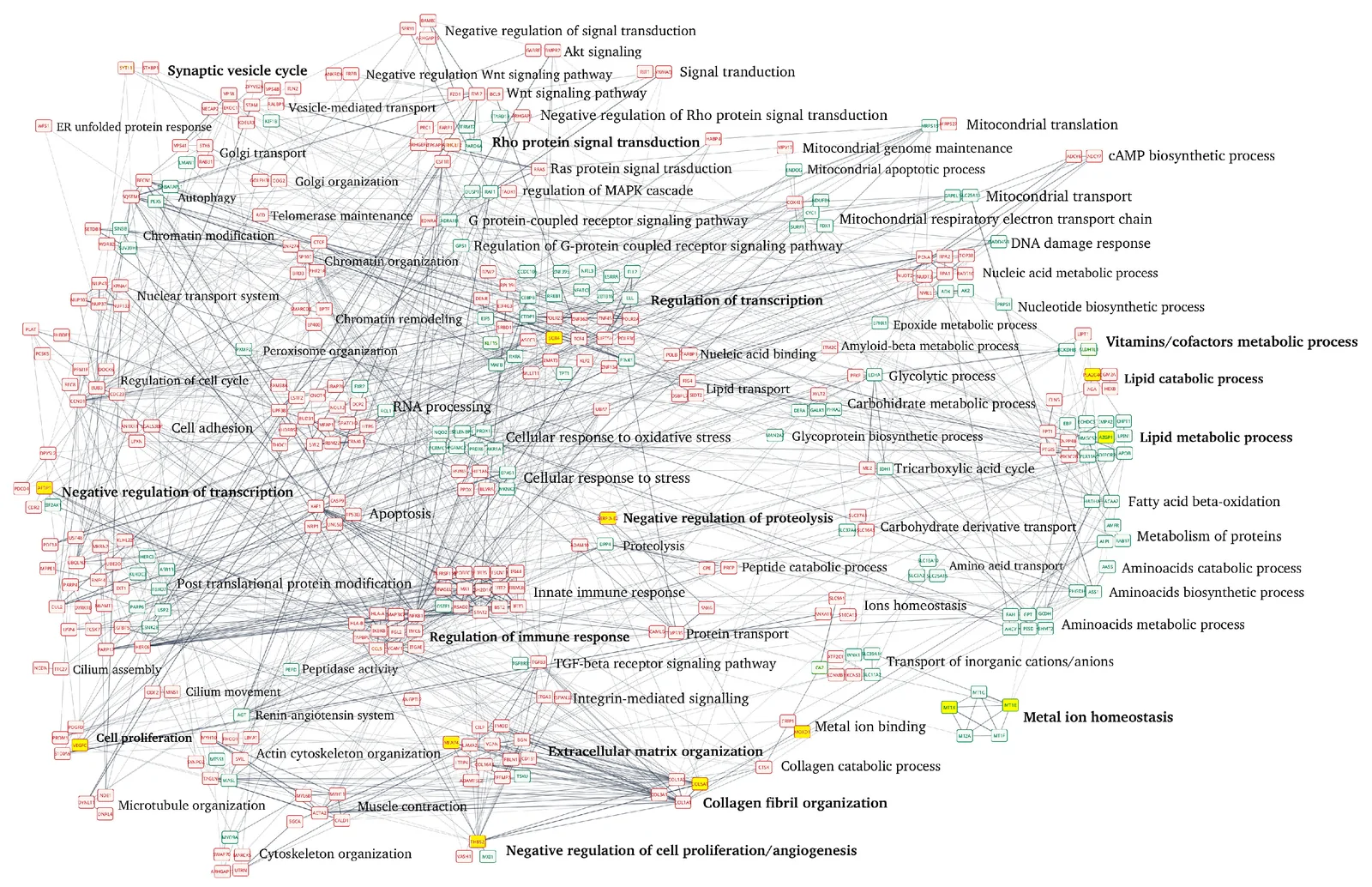
Functional PPI analysis of fibrosis-associated genes. The 12 validated hallmark pan-fibrotic genes are highlighted in yellow. Up-regulated expressed genes are depicted in red, while down-regulated genes are depicted in green. Abbreviations: PPI, protein–protein interaction; ER, endoplasmic reticulum; Akt, AKT serine/threonine kinase; Wnt, Wingless/Integrated; Rho, Ras homologous; Ras, Rat sarcoma; MAPK, mitogen-activated protein kinase; cAMP, cyclic adenosine monophosphate; TGF-β, transforming growth factor beta.

To elucidate functional interactions among the twelve hallmark pan-fibrotic genes, we constructed a protein–protein interaction (PPI) network. The twelve genes were highly interconnected with other molecules previously implicated in fibrosis-associated pathways. Network analysis revealed clustering into distinct modules enriched for pathways related to ECM organisation, signal transduction, immune response, metabolism, regulation of transcription, and metal ion homeostasis (Figure 4).

*THBS2* was the most connected hallmark gene in the network (20 interactions at medium confidence; Table S9). At high confidence it retained five, all within the matrix compartment: the fibrillar collagens *COL1A1*, *COL1A2*, *COL3A1* and *COL5A1*, and the integrin *ITGA9*, placing *THBS2* at the interface between collagen deposition and integrin-mediated cell–matrix adhesion (Table S10). Moreover, direct high-confidence interactions were identified between *CA2* and *CTSK* (0.791) and between *SLC9A1* and *CA2* (0.954; Table S10). At medium confidence, *CTSK* further interacted with *COL1A1*, *COL1A2*, *COL3A1*, *BGN*, *FMOD* and *THBS2*, connecting these genes to the collagen module (Table S9).

In this study, *MT1X* and *MT1E* emerged as novel potential cross-organ fibrosis markers. Within the network at medium confidence, *MT1E* clustered in the “metal ion homeostasis” module together with *MT2A*, *MT1F*, *MT1G* and *MT1X*, and additionally interacted with genes across three clusters, such as the metal-ion transporters *SLC39A14* and *SLC11A2*, and the metabolic genes *APOB* and *GPT*, suggesting that metal ion homeostasis may be connected to transport of inorganic cations/anions and cellular metabolism. At high confidence, the MT module remained connected: *MT1E* and *MT1X* stayed connected to one another and to *MT1F*, *MT1G* and *MT2A*, with *MT1E* retaining the zinc transporter *SLC39A14*. *MT1E* lost its links to the metabolic modules (*APOB*, *GPT*) and to *SLC11A2* (Table S10). The hallmark pan-fibrotic genes were distributed across multiple modules of the network, linking distinct fibrosis-associated pathways (Figure 4; Tables S9 and S10). Collectively, these results indicate that fibrotic progression is accompanied by the coordinated dysregulation of several interdependent pathways, which may therefore represent candidate targets for therapeutic intervention. The inter-module connections were, however, largely confined to the medium-confidence network and should be regarded as hypotheses requiring experimental validation rather than established physical interactions.

### 2.6. Development and Evaluation of the Potential Diagnostic Signatures

**Figure 5:**
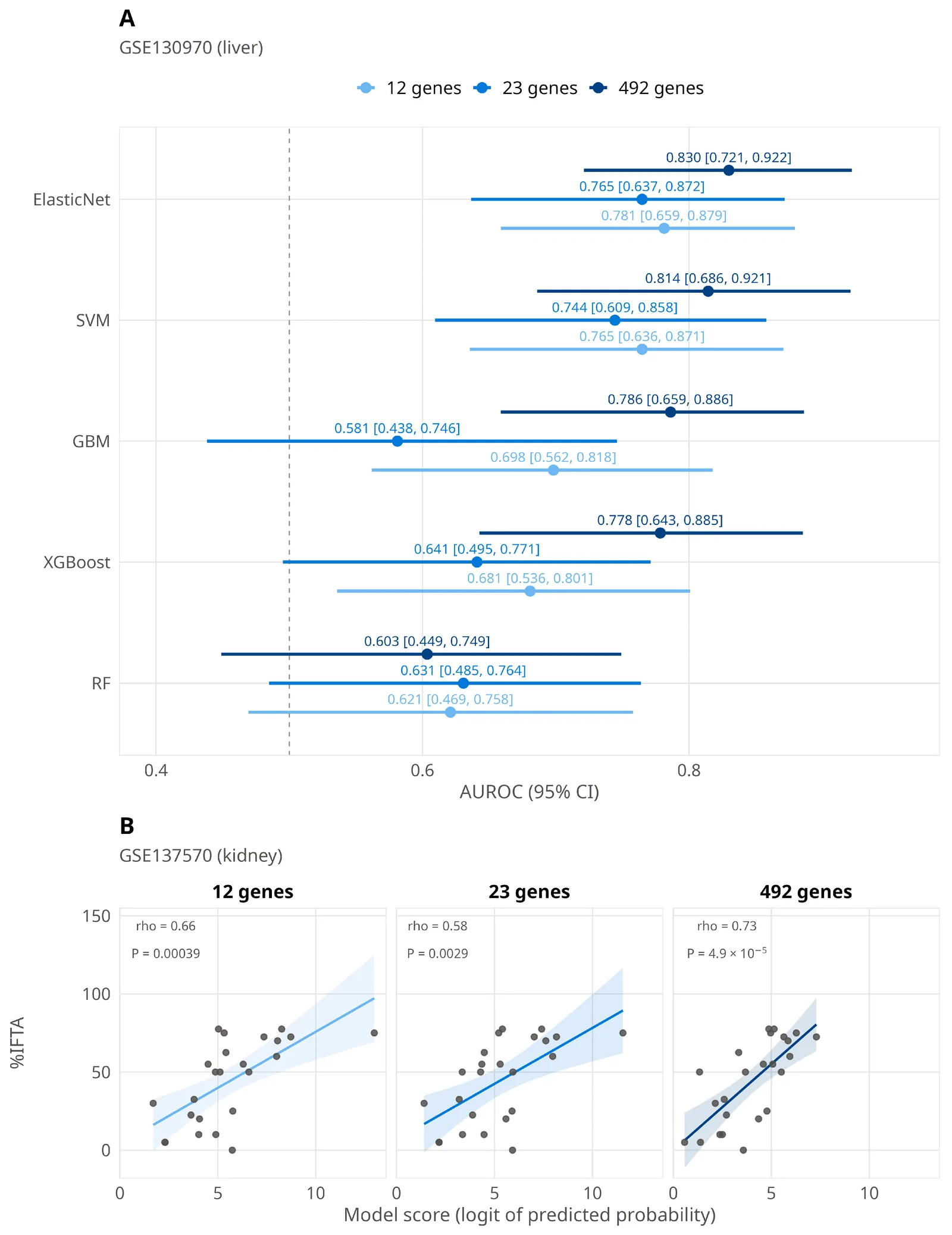
Development and external validation of ML-based diagnostic models using the pan-fibrotic and hallmark gene sets. (**A**) Forest plots showing AUROC values with 95% bootstrap confidence intervals for five ML algorithms (Elastic Net, XGBoost, RF, GBM, and SVM) across three gene sets (g12, g23, and g492) in the liver external validation cohort (GSE130970). The dashed line marks AUROC = 0.5 (**B**) Scatter plots illustrating Spearman’s rank correlation between continuous model scores (logit score of predicted probability) and the %IFTA in the independent kidney validation cohort (GSE137570), across the three gene sets (g12, g23, and g492). The blue line represents the linear regression fit, and the shaded area indicates the 95% confidence interval. Abbreviations: AUROC, area under the receiver operating characteristic curve; CI, confidence interval; ElasticNet, elastic net regularised logistic regression; SVM, support vector machine; GBM, gradient boosting machine; XGBoost, extreme gradient boosting; RF, random forest; %IFTA, percentage of interstitial fibrosis and tubular atrophy; rho, Spearman’s rank correlation coefficient; GSE, Gene Expression Omnibus series accession.

Diagnostic models were developed using five ML algorithms on the 492-gene pan-fibrotic signature and the 23 and 12 hallmark genes. Among the five algorithms evaluated, Enet (α492 = 0.5, α23–12 = 0) achieved the highest discriminative performance for the fibrosis vs. non-fibrosis comparison across all gene sets in the liver validation cohort, with AUROCs of 0.830 (95% CI: 0.721–0.922), 0.765 (95% CI: 0.637–0.872), and 0.781 (95% CI: 0.659–0.879) for the 492-, 23-, and 12-gene models, respectively (Figure 5A; Table S11). Cross-validation selected an Enet penalty performing variable selection (α = 0.5) for the 492-gene model, and a ridge regression (α = 0) for the 23- and 12-gene models. Cross-validated performance at every α tested is reported in Table S12. SVM showed comparable performance, particularly with the larger gene sets (AUROC = 0.814 and 0.744 for the g492- and g23, respectively), and pairwise DeLong tests did not detect significant differences between Enet and SVM for any gene set (all *p* > 0.05). In contrast, the performance differences between Enet and the tree-based methods were more heterogeneous. For the 492-gene model, Enet demonstrated better performance compared to RF (ΔAUROC = 0.226; *p* = 0.008). Moreover, for the 23-gene set Enet demonstrated better performance compared to XGBoost (ΔAUROC = 0.124; *p*-value = 0.03). Consequently, the Enet model was selected for all further analysis. AUPRC was 0.92 (95% CI: 0.84–0.97), 0.89 (95% CI:0.80, 0.95) and 0.90 (95% CI:0.82, 0.96) for the 492-, 23- and 12-gene models, against a cohort prevalence of 0.70. Precision remained at 1.00 over approximately the first quarter of the ranked patients, indicating that every patient in the highest-scoring quartile had histologically confirmed fibrosis (F1–F4) (Figure S2A). This was confirmed by the calibration analysis (Figure S2B). Calibration slopes were close to 1 (0.96–1.08), indicating that the spread of the linear predictor transferred correctly, whereas calibration intercepts ranged from −1.40 to −2.80, indicating systematic over-prediction of absolute risk. After recalibration, calibration intercepts were 0.06, 0.07 and 0.08 and slopes of 0.91, 0.91 and 0.89 for the 12-, 23- and 492-gene models, with Brier scores of 0.169, 0.176 and 0.158, improved relative to the uncalibrated models. Discrimination was essentially unchanged (AUROC 0.767, 0.748 and 0.816, all within 0.02 of the uncalibrated values), as expected. At a probability threshold of 0.5, the 12- and 23-gene models both achieved sensitivity 0.90 (95% CI: 0.78, 0.97), specificity 0.38 (95% CI: 0.18, 0.62), PPV 0.77 (95% CI: 0.64, 0.87) and NPV 0.62 (95% CI: 0.32, 0.86); the 492-gene model achieved sensitivity 0.88 (95% CI: 0.75, 0.95), specificity 0.48 (95% CI: 0.26, 0.70), PPV 0.80 (95% CI: 0.66, 0.89) and NPV 0.62 (95% CI: 0.35, 0.85; Table S13).

In the unadjusted logistic regression analysis, the gene score was significantly associated with fibrosis across all three gene sets (OR_492_ = 2.90 (95% CI: 1.63–5.18), BH *p* = 0.002; OR_23_ = 2.60 (95% CI: 1.45–4.67), BH *p* = 0.003; OR_12_ = 2.95 (95% CI: 1.56–5.60), BH *p* = 0.003). This association remained significant and largely unchanged after adjustment for age and sex (OR_492_ = 2.93 (95% CI: 1.63–5.25), BH *p* = 0.002; OR_23_ = 2.60 (95% CI: 1.44–4.72), BH *p* = 0.003; OR_12_ = 3.00 (95% CI: 1.55–5.80), BH *p* = 0.003).

To further evaluate the clinical relevance of the model scores, their association with fibrosis severity was assessed in the independent kidney validation cohort by computing Spearman’s rank correlation between the continuous model score and the %IFTA. Model scores were significantly and positively correlated with %IFTA across all three gene sets (rho = 0.73, *p* = 4.9 × 10^−5^; rho = 0.58, *p* = 0.0029; and rho = 0.66, *p* = 0.0004 for the 492-, 23-, and 12-gene models, respectively), with the strongest correlation observed for the full 492-gene signature (Figure 5B). These findings indicate that the model scores capture not only fibrosis status but also fibrosis severity.

## 3. Discussion

**Figure 6:**
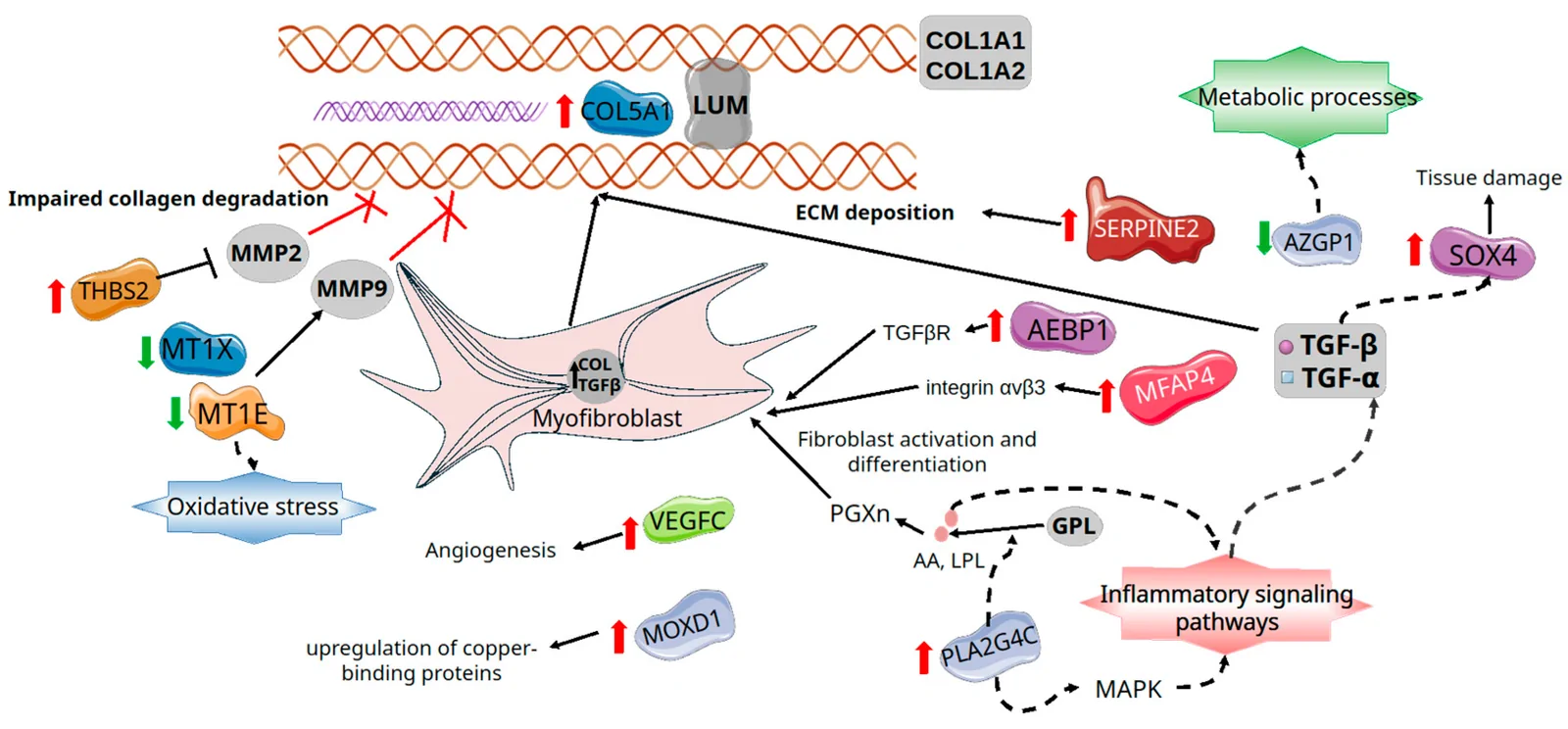
Proposed schematic summary of the hallmark pan-fibrotic genes and their potential functional relevance to fibrosis. The 12 hallmark pan-fibrotic genes identified in this study are shown in colour, while previously known fibrosis-related components (e.g., TGF-β, α-SMA, RhoA/ROCK) are shown in grey for contrast. Red upward and green downward arrows beside each gene indicate, respectively, increased and decreased expression in fibrotic tissue in the present analysis. Solid black arrows denote direct relationships between molecules; dashed black arrows denote indirect or multi-step relationships inferred from the published literature. Bar-headed lines denote inhibition, and red crosses mark processes predicted to be impaired. Abbreviations: AA, arachidonic acid; *AEBP1*, AE binding protein 1; *AZGP1*, alpha-2-glycoprotein 1 zinc-binding; COL, collagen; *COL1A1*/*COL1A2*, collagen type I alpha 1/alpha 2 chain; *COL5A1*, collagen type V alpha 1 chain; ECM, extracellular matrix; GPL, glycerophospholipids; LPL, lysophospholipids; LUM, lumican; MAPK, mitogen-activated protein kinase; *MFAP4*, microfibril associated protein 4; *MMP2*/*MMP9*, matrix metallopeptidase 2/9; *MOXD1*, monooxygenase DBH like 1; *MT1E*/*MT1X*, metallothionein 1E/1X; PGXn, prostaglandins; *PLA2G4C*, phospholipase A2 group IVC; *SERPINE2*, serpin family E member 2; *SOX4*, SRY-box transcription factor 4; *TGF-α*/*TGF-β*, transforming growth factor alpha/beta; TGFβR, transforming growth factor beta receptor; *THBS2*, thrombospondin 2; *VEGFC*, vascular endothelial growth factor C.

Fibrosis is a common pathological endpoint across numerous chronic diseases and a major contributor to progressive organ dysfunction and failure [2]. Despite extensive research into tissue-specific fibrotic mechanisms, few studies have systematically investigated whether shared molecular signatures/pathways exist across different organs [13]. This knowledge gap limits the discovery of broadly applicable pan-fibrotic biomarkers and anti-fibrotic therapies, which could be particularly beneficial in chronic diseases of heart, liver and kidney. In this context, our study aimed to identify a common fibrotic signature through a rigorous, large-scale, multi-cohort, multi-tissue and multi-level transcriptomic analysis covering a total of 2022 unique samples. Specifically, this signature was validated through three sequential, independent platforms, microarray, bulk RNA-seq, and snRNA-seq, resolving cell-type-specific expression of the hallmark genes. Through this integrative approach, we defined a pan-fibrotic gene expression signature comprising 492 genes consistently dysregulated in fibrotic tissues. Furthermore, several of the 23 validated genes demonstrated a significant correlation with fibrosis severity (*ARHGEF2*, *COL5A1*, *PLA2G4C*, *VEGFC*, *CCL5*, and *SOX4*, among others), thereby reinforcing their relevance in disease progression. Among those, twelve were shortlisted as hallmark features, being validated across all data types and organs and their cell-type-specific roles using human snRNA-seq data were described (Figure 6; Table S8). Many of the 12 hallmark genes have been previously associated with fibrosis or are well-established (e.g., *COL5A1*, *VEGFC*, *SERPINE2*, *THBS2*, *AEBP1* and *AZGP1*), supporting the validity of our cross-organ and cross-platform approach in capturing known fibrotic components. In contrast, some of these hallmark genes including MT1E, *MT1X*, *MOXD1*, *PLA2G4C*, and *SOX4* have not been previously reported as fibrosis-associated in humans across different organs, providing additional insights into conserved pathophysiological mechanisms of fibrosis.

Previous studies have demonstrated that the development of fibrosis is, in part, associated with excessive collagen production [2,13,14]. In line with this, our analysis identified altered expression of a heterogeneous set of fibrosis-associated genes, including ECM structural and matricellular components (*COL5A1*, *MFAP4*, *THBS2*), signalling molecules (*VEGFC*, *SERPINE2*), transcription factors (*SOX4*, *AEBP1*), metal-binding and lipid metabolic proteins (*MT1E*, *MT1X*, *PLA2G4C*), an oxidative stress regulator (*MOXD1*), and the protective factor *AZGP1*.

The transcription factor *SOX4* was upregulated in fibrotic tissues. *SOX4* belongs to the SOX family of transcription factors, which play important roles in tissue morphogenesis, development, and various disease processes [15]. Although *SOX4* has not been extensively studied in the context of fibrosis across multiple organs, emerging evidence suggests its potential relevance: in cardiac pathology, *SOX4* expression correlates with fibrotic gene signatures in both dilated and hypertrophic cardiomyopathy, suggesting its involvement in this process [16]. Furthermore, in a liver injury model, *SOX4* expression was induced by injury-associated signals such as TGF-β, Notch, YAP, contributing to pathological remodelling and tissue damage [15]. Interestingly, the latter regulatory response parallels the dynamics observed in SOX9-driven regeneration, where its sustained activation determines whether tissue regeneration proceeds adaptively or results in fibrosis [17].

We observed consistent upregulation of *PLA2G4C*, a member of the phospholipase A2 (PLA2) superfamily of lipolytic enzymes. *PLA2G4C* catalyses the hydrolysis of glycerophospholipids, leading to the release of free fatty acids such as arachidonic acid and lysophospholipids. This reaction represents a step in the biosynthesis of pro-inflammatory prostaglandins, which have been implicated in fibroblast activation and differentiation, thereby contributing to fibrotic remodelling [18]. Furthermore, different secretory *PLA2* isoforms were found upregulated in fibroblast of patients with Idiopathic Pulmonary Fibrosis (IPF) and treatment with a known sPLA2 inhibitor attenuated lung fibrosis along with a reduction in *TGF-β* and deposition of ECM in lung [19].

A novel finding of our study was the identification of *MT1E* and *MT1X*, members of the MT family, as consistently downregulated across all tissues and negatively correlated with fibrosis severity, together with alterations in pathways related to MT-bind metals, zinc ion homeostasis and cellular response to zinc ion (Tables S4 and S8). These findings suggest that impaired intracellular zinc homeostasis may represent an underexplored common mechanism in fibrosis. Notably, the primary transmembrane zinc transporter *SLC39A14* was also downregulated across all tissues (Table S1). Given that *SLC39A14* mediates cellular zinc uptake, its reduced expression could contribute to the suppression of MT via reduced intracellular zinc availability [20,21]. Other MT isoforms (*MT2A*, *MT1F*, and *MT1G*) showed similar trend of downregulation (although not reaching statistical significance across all tissues), further supporting the findings (Table S1). To our knowledge, this represents the first report of consistent downregulation of MTs and *SLC39A14* in association with fibrosis progression across multiple human organs, highlighting their potential as novel, cross-organ-fibrosis markers and/or regulators. This finding is consistent with independent pre-clinical and clinical evidence linking zinc imbalance to fibrosis affecting the heart, kidney and liver [22,23,24,25], as well as independent pre-clinical literature evidence in *Slc39a14* knockout mice, which exhibit reduced zinc levels and lower Mt-1 expression [26]. Notably, in murine liver fibrosis models, zinc supplementation restored *Slc39a14* expression and reversed fibrotic process [27], reinforcing the role of these genes as mediators of zinc homeostasis in fibrosis progression. Together, these findings support a proposed mechanistic hypothesis linking SLC39A14-mediated zinc transport, MT expression, and MMP9-dependent ECM degradation. Nevertheless, further research is required to provide direct confirmation of this pathway in human fibrotic tissue.

Although direct links between *MT1E* and *MT1X* isoforms and fibrosis or ECM remodelling are limited, prior studies in cell lines have shown that silencing of related isoform *MT2A* in cell lines reduces MMP9 activity [28,29]. This observation suggests that *MT1E* and *MT1X* downregulation, together with reduced zinc ion availability, may be associated with impaired MMP9 catalytic function and reduced ECM degradation [25,28,29,30]. However, further experimental validation, such as direct enzymatic assays, is required to confirm these findings. Notably, in our study several MMPs showed increased expression in fibrotic tissues, potentially reflecting a compensatory transcriptional response to support matrix degradation (Table S1). We hypothesise that their enzymatic activity may be compromised in a zinc-deficient environment, although MMP activity was not measured in the present study. Concurrent upregulation of *TIMP1* (as indicated by our results) in this environment may further inhibit MMP function, thereby in part contributing to impaired resolution of fibrosis [27] (Table S1). These findings are consistent with previous in vivo studies: in a mouse model of intermittent hypoxia, MT silencing results in increased aortic fibrosis [31]. Conversely, MT-overexpressing transgenic (MT-TG) mice demonstrated protection against progressive myocardial fibrosis during the long-term follow-up (1 to 6 months) and against liver fibrosis [32,33]. Taken together, our findings suggest a potential anti-fibrotic role for *MT1E* and *MT1X*, likely through their involvement in regulating MMP-dependent ECM degradation. We emphasise that this proposal is based on transcript-level associations in our data, combined with published functional evidence from other systems. Neither MT protein abundance, tissue zinc concentration nor MMP enzymatic activity was measured in this study. While fibrillogenic genes are broadly expressed across tissues and contribute to excessive collagen production, the consistent downregulation of MT and zinc homeostasis pathways points to an additional, complementary mechanism: impaired ECM degradation. Together, these data support the notion that fibrosis is the result not only of increased ECM synthesis, but also of attenuated matrix remodelling and degradation.

Beyond gene-level findings, our study identifies dysregulated biological processes that are conserved across fibrotic organs. Among the activated pathways, those related to ECM remodelling and integrin signalling, as well as previously described core signalling pathways such as Wnt/β-catenin signalling and fibroblast growth factor [2,9,10], were predominant. This finding underscores the central role of structural reorganisation in fibrosis. Pathways that were consistently inhibited across fibrotic organs were related to metallothioneins bind metals, zinc homeostasis, metabolic processes, mitochondrial metabolism and transcriptional regulation (Table S4). These findings are in line with recent evidence linking metabolic reprogramming to chronic diseases, in which changes in cellular metabolism have been associated with myofibroblast activation and improved collagen stabilisation [34,35]. Overall, our results indicate that metabolic dysfunction, together with disturbed metal ion regulation, represents a complementary layer of dysregulation that may contribute to shaping the fibrotic phenotype across tissues.

Interestingly, the PPI network analysis further demonstrated that several hallmark pan-fibrotic genes act as key nodes, linking different fibrotic processes including ECM organisation, immune signalling, metabolic activity, and metal ion homeostasis. These findings reflect the complex nature of fibrosis, indicating that its progression involves the coordinated disruption of multiple, interdependent biological systems rather than isolated pathways. *THBS2* was the most connected hallmark gene, retaining at high confidence interaction with the fibrillar collagens *COL1A1*, *COL1A2*, *COL3A1* and *COL5A1* and with the integrin *ITGA9*, placing it at the interface between collagen deposition and integrin-mediated adhesion. Importantly, some pan-fibrotic genes not previously associated with fibrosis, such as *MT1E*, *MT1X*, *MOXD1*, *SOX4*, and *PLA2G4C* were observed across distinct pathways, suggesting possible roles in mediating cross-pathway communication during fibrotic remodelling.

A further novel element of our study was the cell-type-resolved investigation of the pan-fibrotic signature in human tissues: snRNA-seq analysis revealed that the proportion and transcriptional activity of cell populations shifted across fibrosis stages, revealing a more complex cellular architecture than previously described. Although in previous studies HSCs were well-established as key mediators of fibrosis due to their central role in ECM production [36,37], our findings indicate that other hepatic cell types actively contribute to fibrosis progression. Specifically, we observed that hallmark genes involved in ECM production and remodelling were upregulated in a cell-type-restricted manner, with *VEGFC* and *COL5A1* altered in endothelial cells, *SOX4* and *AZGP1* in hepatocytes; *MOXD1* significantly altered in HSCs. The expression of *MT1E*/*MT1X* was reduced in hepatocytes, HSCs and LSECs in the severe vs. non-fibrosis comparison, with a significant reduction observed at the tissue level. These differential expression patterns suggest that multiple hepatic cell types contribute to different components of fibrosis. This pattern is consistent with the well-described perisinusoidal fibrotic niche, in which scar-associated mesenchymal cells, HSCs, endothelial cells, and infiltrating immune cells interact within the stiffened matrix, thereby promoting fibrosis [38]. Given the conservation of the signature, we hypothesise that analogous fibro-immune-endothelial interaction patterns underlie fibrotic remodelling in the heart and kidneys too. In fact, spatial profiling in heart, kidney and liver tissues, indicated a conserved intercellular communication mechanism across associated mesenchymal cells, endothelial cells, and infiltrating immune cells, driving fibrotic remodelling [39,40,41]. The cell-type-resolved expression patterns identified here provide a molecular foundation for future spatial profiling studies, by identifying specific cell types and hallmark genes as priority targets for spatial investigation across organs. Future multi-organ spatial and multi-omics studies will be required to further define interaction networks centred on the hallmark genes and niche organisation across fibrotic organs. In addition, studies focusing on dynamic biological processes underlying transitional cell states will be essential to elucidate how the identified hallmark genes are regulated as cells transition between distinct activation states.

In the present study, we analysed the pan-fibrotic and hallmark signature in two independent fibrotic cohorts, thereby establishing a potential diagnostic model for fibrosis. The Enet and SVM consistently demonstrated the strongest performance, with the 492-gene signature yielding the highest AUROCs in external validation. Moreover, significant correlation between Enet model scores and %IFTA in the independent kidney cohort demonstrates cross-organ generalizability and the ability to capture a continuous fibrotic pattern. In this context, the pan-fibrotic signature is intended to prioritise candidate therapeutic targets that are applicable across organ systems, and in providing a tissue-level molecular reference to guide the development and interpretation of non-invasive assays. Translating the hallmark genes identified here into analytes measurable in accessible biofluids, together with a formal evaluation of the cost-effectiveness and assay requirements of any resulting test, will be necessary steps before clinical application.

Our study has several limitations arising from the analysis of publicly available data. Firstly, the discovery cohort included patients with chronic diseases known to involve fibrosis, but the detailed metadata on fibrosis was not available. However, this was addressed through rigorous validation across multiple platforms and independent cohorts with comprehensive fibrosis-stage annotation, significantly strengthening reproducibility. Secondly, the microarray discovery and initial validation cohorts share the same 140 control samples, reflecting the limited availability of histologically normal liver tissue in public repositories. The fibrotic cases of the two cohorts are nonetheless entirely independent, and the sensitivity analyses confirmed the hallmark signature with unchanged effect sizes (Tables S2 and S3). Reproducibility is further supported by the bulk RNA-seq and snRNA-seq validation cohorts, which share no samples with the discovery analysis. In addition, a subset of the pan-fibrotic and hallmark signature may also reflect chronic organ injury rather that fibrosis specifically. However, the specificity of the signature for fibrosis is further supported by the identification of the majority of hallmark genes showing significant correlation between expression and continuous fibrosis severity in the RNA-seq validation cohorts (15 of 19 genes; Figure 2C–F and Figure S1), by the significant correlation of composite model scores derived from the 492-, 23-, and 12-gene sets with %IFTA in an independent kidney validation cohort (Figure 5B), and by pathway-level analyses further identifying ECM assembly, collagen fibril organisation, and related structural remodelling processes over generic injury signalling (Figure 1B–D). Thirdly, although snRNA-seq datasets may be affected by sample dissociation-related biases [42], this was mitigated by focusing on snRNA-seq datasets, which exhibit minimal dissociation-induced alterations [43,44,45], and by basing discovery and validation analysis on bulk transcriptomic data derived from intact tissue. Furthermore, the identification of well-known fibrosis-associated genes within the pan-fibrotic gene expression signature across multiple tissues and platforms, along with the identification of all major hepatic cell populations in the integrated snRNA-seq analysis, supports the reliability and validity of the datasets used. Fourthly, the present study does not include direct experimental validation in animal models or cell lines, and functional studies, such as knockdown or knockout of *MT1E* and *MT1X*, direct assessment of MMP enzymatic activity by zymography, and measurement of MT protein abundance and tissue zinc concentrations in fibrotic tissue, are required to determine the causal role of the pan-fibrotic signature and to confirm the proposed zinc-dependent mechanism of impaired ECM degradation. Fifthly, the pan-fibrotic signature is derived entirely from transcriptomic data. Analysis of proteomic datasets would be required to confirm that the identified genes are consistently altered at the protein level across organs. Sixthly, while the ML-based diagnostic models demonstrated promising performance, the additional validation cohorts were relatively small, particularly the kidney cohort (*n* = 24), which limits conclusions regarding generalizability and the precision of the estimation of discrimination. Consequently, the primary analysis in the kidney cohort was based on the Spearman correlation between the continuous model score and %IFTA; larger prospective cohorts will be needed to confirm clinical applicability. Nevertheless, the translational relevance of our findings is reinforced by the large-scale, multi-organ integration analysis of thousands of human samples across multiple datasets and tissues. Additionally, existing literature from animal and cell-line models, as highlighted in our discussion, supports our findings and underscores the potential of the identified genes, thus paving the way for future pre-clinical and mechanistic studies.

## 4. Materials and Methods

### 4.1. Study Design and Dataset Retrieval

**Figure 7:**
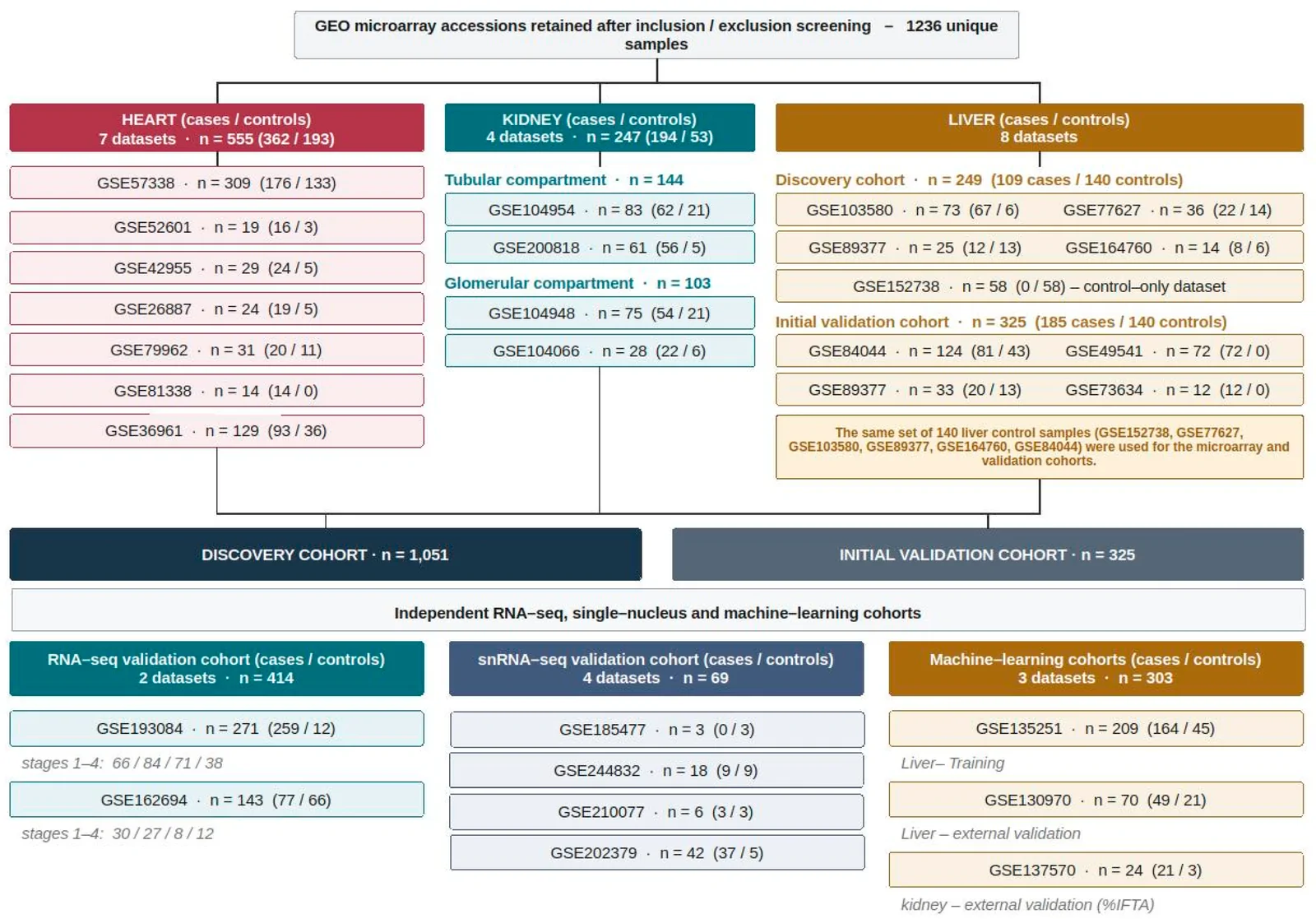
Data flow summarising the per-dataset and per-cohort composition of all microarray, RNA-seq, single-nucleus RNA-seq (snRNA-seq), and machine learning (ML) datasets included in this study. For each dataset, sample counts are reported as *n* (cases/controls); fibrosis-stage distributions across stages 1–4 are additionally indicated for the RNA-seq validation cohorts.

To investigate the conserved molecular fibrotic signature across various organs, publicly available transcriptomics and clinical data acquired from the liver, kidney, and heart tissues, obtained from patients diagnosed with chronic disease involving fibrosis, were retrieved from Gene Expression Omnibus (GEO, https://www.ncbi.nlm.nih.gov/, accessed on 21 December 2022) database, and analysed through a multi-step and cross-platform process, as depicted in Figure 7. Details on the systematic data retrieval and curation of both publicly available microarray, and RNA-seq data are presented in Figure S3. In order to validate the hallmark genes, a part of the RNA-seq data was utilised, while the remainder was employed for the training and validation of ML model.

A total of 1236 unique microarray samples from human tissue samples (specifically from heart, kidney and liver) were retrieved, including GSE57338 [46], GSE52601 [47], GSE42955 [48], GSE26887 [49], GSE79962 [50], GSE81338 [51], GSE36961 [52], GSE104954 [53], GSE200818 [54], GSE104948 [55], GSE104066 [56], GSE152738 [57], GSE77627 [58], GSE103580 [59], GSE89377 [60], GSE164760 [61], GSE73634 [62], GSE84044 [63], and GSE49541 [64]. Due to limited availability of information about the stage of fibrosis, microarray datasets were divided into 2 groups: a discovery cohort comprising studies of chronic heart (*n* = 555 samples), kidney tubules (*n* = 144 samples), kidney glomeruli (*n* = 103 samples) and liver diseases (*n* = 249 samples) known to be associated with fibrosis (*n* = 1051; Tables S14–S16) and validation cohort with available fibrosis staging data (*n* = 325; Table S17). Fibrosis was defined histologically in the liver discovery datasets, in which all cases were diagnosed as cirrhosis and controls as histologically normal liver; samples lacking information on fibrosis were excluded during curation (Table S16). In the heart and kidney datasets, where per-sample histological grading was unavailable, fibrosis was inferred at the disease level from end-stage cardiomyopathy and chronic kidney disease, respectively. Both of these conditions are characterised by established interstitial fibrosis. The same set of 140 liver control samples (GSE152738, GSE77627, GSE103580, GSE89377, GSE164760 and GSE84044) were used in both the discovery and the initial validation microarray cohorts, due to the limited availability of histologically normal liver tissue information in public repositories. The combination of the available controls increased the statistical power of both analyses. None of these 140 samples is present in any downstream cohort: the bulk RNA-seq validation datasets (GSE193084, GSE162694), the snRNA-seq datasets (GSE185477, GSE244832, GSE210077, GSE202379) and the ML training and test cohorts (GSE135251, GSE130970, GSE137570) are independent studies contributing their own controls. The consequences of this control overlap were assessed in a dedicated sensitivity analysis (see Section 4.4). A complete per-dataset assignment, with sample counts, organ compartment and cohort, is provided in Figure 7. Moreover, 414 liver tissue samples from two RNA-seq datasets, including GSE193084 [65] and GSE162694 [66], with detailed fibrosis stage information were acquired and included as a second independent validation cohort (nRNAseq1 = 271, nRNAseq2 = 143; Table S18).

Four publicly available snRNA-seq datasets of human liver tissue (*n* = 69) covering different fibrosis stages, including GSE185477 [67], GSE244832 [68], GSE210077 [69], and GSE202379 [70], were acquired as part of the validation process (Table S19). The analysis focused specifically on liver fibrosis, as these datasets included samples with annotated fibrosis stages determined by histological assessment of routine staining, as described previously in [71,72,73]. Given the heterogeneity in fibrosis staging systems across studies, liver fibrosis scores were harmonised into low, moderate, and severe categories based on standardised histological classifications (Table S20) [74,75,76,77,78]. To train and validate the ML algorithms, gene expression and clinical information of RNA-seq were retrieved from multiple cohorts, including GSE135251 [79], GSE130970 [80], and GSE137570 [81] (Table S21).

### 4.2. Bulk Datasets Processing

All raw microarray data were uniformly retrieved, processed, and then integrated within each organ system, following the previously described pipeline [82]. Only microarray studies with at least 10 samples were retained for analysis, to ensure sufficient power and robustness of the differential expression results. Raw files of each GEO dataset were individually pre-processed applying background correction, quantile normalisation and log transformation of expression values. Individual datasets were merged per tissue, except for kidney tissue, where tubular and glomerular compartments were analysed separately. This integration was achieved through common genes and to ensure comparability across the different GEO datasets within the organ system. Batch effect removal was applied to gene expression data using the ComBat algorithm [83]. This adjustment was evaluated using relative log expression and principal component analysis plots, supporting the successful removal of the batch effect (Figures S4–S7). Importantly, biological variability was preserved, as evidenced by the sustained high expression levels of heart-specific housekeeping genes (*CASQ2*, *CRYAB*, *UBB* and *MYL3*) in the merged heart tissue dataset (Figure S8).

For the analysis of bulk RNA-seq data, the two GEO single-end RNA-seq datasets of liver tissue were considered as a second validation cohort and analysed individually. Raw FASTQ files were aligned to the human reference genome sequence (hg38) using STAR with default parameters, and the package Rsubread (v1.5) with the featureCounts function was used to generate summary counts. Genomic alignment was found to be consistently high across all 732 bulk RNA-seq samples, with a mean uniquely mapped rate of 86.6% ± 7.1% (range 62.1–95.6%), and no sample falling below 60%. Samples with a successfully assigned rate below 55%, that is the proportion of reads assigned by featureCounts to annotated exonic features, were excluded from further analysis; this criterion was applied to all bulk RNA-seq datasets analysed in this study, including the ML cohorts. RNA-seq data were analysed per dataset to further increase confidence in the reproducibility of the findings.

### 4.3. SnRNA-Seq Data Retrieval and Processing

Analysis of snRNA-seq data was restricted to liver tissue, as no stage-annotated heart or kidney datasets were identified at the time of the search. Fibrosis-stage metadata was a required inclusion criterion, as it enabled validation of the pan-fibrotic signature against disease severity. SnRNA-seq datasets were retrieved through a PubMed search using the keywords “liver fibrosis,” “NAFLD,” “NASH,” “cirrhosis,” and “hepatitis,”. Studies with available metadata detailing fibrosis stage and with public access to raw or processed snRNA-seq data were included. Those datasets involving infectious diseases or liver cancer were not considered for the analysis. To minimise integration bias and ensure methodological consistency across studies, only snRNA-seq datasets were included, while scRNA-seq datasets were excluded. This choice also minimises potential artefacts introduced by conventional enzymatic digestion, which can under-represent matrix-embedded or structurally constrained cells, and therefore is particularly suited to the study of fibrosis-related changes in tissue architecture.

Data processing and downstream analysis were performed using the Python package Scanpy (v1.11.0). Raw count matrices were obtained and loaded into Scanpy objects for quality control filtering. Cells expressing fewer than 500 or more than 5000 genes, or exhibiting over 10% mitochondrial transcript content, were excluded. After quality filtering, the resulting AnnData objects from individual samples were merged into a single dataset for subsequent integration and analysis. A total of 249,088 high-quality cells with 17,684 genes were retained. To perform dimensionality reduction, batch correction, and clustering, the top 4000 highly variable genes across batches were identified. The batch effect arising from diverse platforms and protocols was subsequently removed using 50 principal components (PCs) with the BBKNN algorithm from the Python package BBKNN (v1.6.0). Clustering in the integrated object was performed using the Leiden algorithm with parameter resolution = 3, and clusters were visualised using Uniform Manifold Approximation and Projection (UMAP) with parameters min_dist = 0.1 and spread = 1.4. Cell clusters were manually annotated according to the expression of canonical marker genes for each cluster (Table S22).

### 4.4. Defining the Pan-Fibrotic Gene Signature

To define pan-fibrotic gene expression signature based on the discovery microarray data, statistical analysis was first conducted for each organ system based on the integrated ComBat corrected data. Genes that were statistically significant and showed consistent fold change directionality in the individual datasets compared to the integrated data within each organ, were retained. Subsequently, to identify the pan-fibrotic gene expression signature, differential expression between fibrotic and non-fibrotic samples was assessed within each organ on the Combat-corrected integrated data using the Wilcoxon rank-sum test, with BH correction. Genes with an adjusted *p* < 0.05 were considered significant. No fold-change threshold was applied, and both up- and downregulated genes were retained. The directionality of the change for each signature gene was further verified using independent microarray-derived dataset with known information about the fibrosis stage.

To further shortlist the key components of the pan-fibrotic gene expression signature, significance across all organ systems was required. This was followed by validation of both the direction of regulation and statistical significance in two bulk RNA-seq datasets and an integrated snRNA-seq dataset. A sensitivity analysis was performed to determine whether the reproducibility of the pan-fibrotic signature depended on the control samples shared between the discovery and initial validation microarray cohorts. Liver differential expression analyses were recomputed on the ComBat matrix in the two cohorts with no overlapping controls, using the Wilcoxon rank-sum test with BH correction. The discovery cohort comprised 109 cases and 84 controls (GSE152738, GSE77627, GSE103580 and GSE164760), while the validation cohort comprised 185 cases and 56 controls (GSE84044 and GSE89377). Agreement between the two cohorts was assessed by the direction of change, by significance at BH *p* < 0.05 in both and in at least one cohort, and by the Pearson correlation of log2 fold changes with those genes identified in the primary analysis.

### 4.5. Pathway Enrichment Analysis

Pathway activation scores in the bulk transcriptomics data were calculated using the pan-fibrotic gene expression signature (492 genes) through single-sample gene set enrichment analysis (ssGSEA) as implemented in the GSVA R/Bioconductor package (v1.50.5). The analysis was performed separately for each organ system, based on the ComBat corrected integrated microarray discovery, as well as on the RNA-seq validation datasets. Pathway analysis of the RNA-seq data was conducted separately for each dataset using all respective significantly affected genes identified per dataset (BH *p* < 0.05). Gene libraries from Hallmark (v7.5.1), Canonical Pathways (Reactome subset, v7.5.1), Gene Ontology (GO, BP, CC, MF v7.5.1), and Transcription Factor Targets (GTRD, v7.5.1), were retrieved from the Molecular Signature Database (MSigDB) using the R software (v4.1.0). Pathway concordance was defined as consistent enrichment (BH *p* < 0.05) with the same direction of activation status (activation or inhibition) across all fibrosis-affected organ systems in the discovery and initial validation cohort. Subsequently, these fibrosis-associated pathways were verified using the RNA-seq datasets. Those pathways found to be significantly enriched (BH *p* < 0.05) in each RNA-seq dataset and maintained the same activation or inhibition status in the microarray comparisons were considered as verified.

### 4.6. Functional Protein–Protein Interaction Network

In order to explore the functional connectivity and potential regulatory hubs among the hallmark signature (12 genes), a PPI network was generated using the STRING database (v2.2.0) and visualised with Cytoscape software (v3.10.3) [84], with interaction scores set at a medium and high confidence threshold (≥0.4 and ≥0.7). The analysis was based on 12 pan-fibrotic genes and other components within the activated and deactivated pathways identified in the discovery and initial validation cohort in which these shortlisted genes were involved. Moreover, to assess the functional context of genes within the PPI network, a manual assignment based on a literature review of biological processes associated with each gene was performed. Genes were grouped into clusters that reflect shared biological processes.

### 4.7. Machine Learning Models and Validation

For each predefined gene signature (492, 23, and 12 genes), separate binary classifiers were trained to evaluate their diagnostic potential for fibrosis, using five supervised learning algorithms: random forest (RF), extreme gradient boosting (XGBoost), gradient boosting machine (GBM), elastic net (Enet), and support vector machine (SVM). Raw counts were normalised using Trimmed Mean of M-values (TMM) and transformed to log2-Counts Per Million (CPM). Hyperparameters were tuned exclusively within the training cohort using stratified 5-fold cross-validation repeated 20 times, with class-balanced weighting applied to mitigate class imbalance. For the Enet models (glmnet v4.1.10), the α parameter was tuned over the grid {0, 0.25, 0.5, 0.75, 1} with fold partitions held identical across candidate α values and selected on maximum mean cross-validated AUROC. Discrimination in external cohorts was assessed by the area under the receiver operating characteristic curve (AUROC) with 95% bootstrap confidence intervals (2000 iterations). To assess association with fibrosis severity in kidney, Spearman’s rank correlation was computed between the model score (logit score of predicted probability) and the %IFTA in GSE137570. Classifiers were developed in GSE135251 to discriminate F1–F4 from F0 and evaluated in GSE130970 (liver) and GSE137570 (kidney). Five algorithms were fitted (Enet, gradient boosting, random forest, support vector machine, XGBoost) on three gene sets (492, 23, 12 genes). Counts were TMM-normalised within each cohort separately. Standardisation parameters were estimated on training data only and applied unchanged to validation data and were re-estimated within each cross-validation fold on that fold’s training portion. Class weights were derived from the training subset. Hyperparameters were selected by repeated stratified cross-validation in the training cohort, and the classification threshold was fixed on training data at 90% specificity. The AUROC and AUPRC were reported against cohort prevalence, with 95% bootstrap CI. Calibration was assessed using the intercept and slope of logistic regression of the outcome on logit-transformed predicted probabilities and the Brier score. Sensitivity, specificity, PPV and NPV are reported with exact (Clopper-Pearson) 95% intervals. Because external predicted probabilities showed systematic over-prediction with a calibration slope near 1, we additionally performed logistic recalibration of the intercept and slope (Platt scaling). In this process, the fitted model remained unchanged, and only the α and β parameters were estimated using a 5-fold, stratified cross-validation approach repeated 20 times within the GSE130970 dataset.

### 4.8. Statistical Analysis

Statistical analysis of the bulk transcriptomics data was performed using the R software (v4.1.0), including the stats package for the Wilcoxon rank-sum test (for microarray), the DESeq2 package for the negative binomial Wald test (for RNA-seq), and the stats package for Spearman’s correlation. False discovery rate (FDR) correction was applied using the BH method, an adjusted *p* < 0.05 was considered statistically significant. In all cases, comparisons were made between “fibrotic” and “non-fibrotic” conditions. Expression of the hallmark genes across fibrosis stages was assessed in the RNA-seq validation cohorts using the Kruskal–Wallis test as a global test of difference across the five ordinal fibrosis stages (0–4), followed by pairwise comparisons using the two-sided Wilcoxon rank-sum test. All tests were implemented using the ggpubr R package (v0.6.2).

In the snRNA-seq data, differential expression was assessed at the patient level. Ambient RNA was first removed using the DecontX algorithm from the celda R/Bioconductor package (v1.18.2), applied separately to each dataset with the patient as batch and the annotated cell type as cluster label. Decontaminated counts were then summed across nuclei within each patient and cell type, giving one expression profile per biological replicate. Profiles were normalised using the TMM method from the edgeR R/Bioconductor package (v4.0.16), weighted by voom, and fitted with a linear model of the form ~ dataset + stage using the limma R/Bioconductor package (v3.58.1), with “no fibrosis” as the reference level and dataset of origin included to account for the four cohorts. Low, moderate and severe fibrosis were each compared with no fibrosis using moderated t-statistics. Because the hallmark genes were pre-specified from the previous microarray and bulk RNA-seq analyses, BH correction was applied across those genes within each cell type and contrast. A patient × cell type profile was retained when it comprised at least 10 nuclei, and a comparison was evaluated only when at least five patients remained in both groups after this filter. Cell type proportions were computed per patient, transformed by arcsine square root, and compared across fibrosis stages using limma with the dataset included as a covariate; results were confirmed under a logit transformation. All analyses were performed using the R software (v4.3.3).

## 5. Conclusions

In summary, by integrating multi-organ transcriptomic data from the heart, liver, and kidney, this study defined a pan-fibrotic gene expression signature and shared regulatory pathways, highlighting potential new molecules implicated in fibrosis across organs (e.g., *MT1E*, *MT1X*, *PLA2G4C*, *MOXD1*, and *SOX4*), thus providing additional insights into the molecular mechanisms underlying this pathological process. At the single-cell level hallmark genes (such as ECM genes) are significantly differentially expressed in hepatocytes and endothelial cells, with *MOXD1* altered specifically in HSCs. Collectively, these results not only verify known disease mechanisms but also highlight novel potential candidates for targeted functional studies. Further, ML-based diagnostic models trained on the pan-fibrotic and hallmark gene sets demonstrated promising discriminative performance in independent validation cohorts, with model scores independently associated with fibrosis outcome after adjustment for clinical covariables and significantly correlated with histological fibrosis severity across organ systems. These insights underscore the need for multi-targeted therapeutic approaches and lay the groundwork for cross-organ, precision medicine strategies in fibrosis treatment.

## Data Availability

The datasets analysed during the current study are publicly available data in GEO repository: GSE57338, GSE52601, GSE42955, GSE26887, GSE79962, GSE81338, GSE36961, GSE104954, GSE200818, GSE104948, GSE104066, GSE152738, GSE77627, GSE103580, GSE89377, GSE164760, GSE73634, GSE84044, GSE49541, GSE193084, GSE162694, GSE185477, GSE244832, GSE210077, GSE202379, GSE135251, GSE137570, GSE130970. The code supporting the findings of this study has been deposited at Zenodo via: https://doi.org/10.5281/zenodo.15799000.

## Supplementary Materials

The following supporting information can be downloaded at: https://www.mdpi.com/article/10.3390/ijms27198755/s1.
