# Supplementary material for: Pan-Fibrotic Gene Expression Signature in Major Chronic Diseases by Integrative Bulk and Single-Cell Transcriptomic Analyses
Source: Int J Mol Sci. 2026 Sep 30;27(19):8755. doi: 10.3390/ijms27198755 (PMC13665512; doi:10.3390/ijms27198755)
Supplement: Supplementary file 1 [file ijms-27-08755-s001.zip › ijms-4556196_supplementary_clean_290926.pdf]

Supplementary material

Pan-fibrotic gene expression signature in major chronic diseases by integrative bulk and single-cell transcriptomic analyses

Mayra Alejandra Jaimes Campos, Rafael Stroggilos, Joost-Peter Schanstra, *Antonia Vlahou*, Manon Brunet*, Jean-Christophe Jonas, Mohammed Bensellam, Harald Mischak, Agnieszka Latosinska*

Supplementary Figures

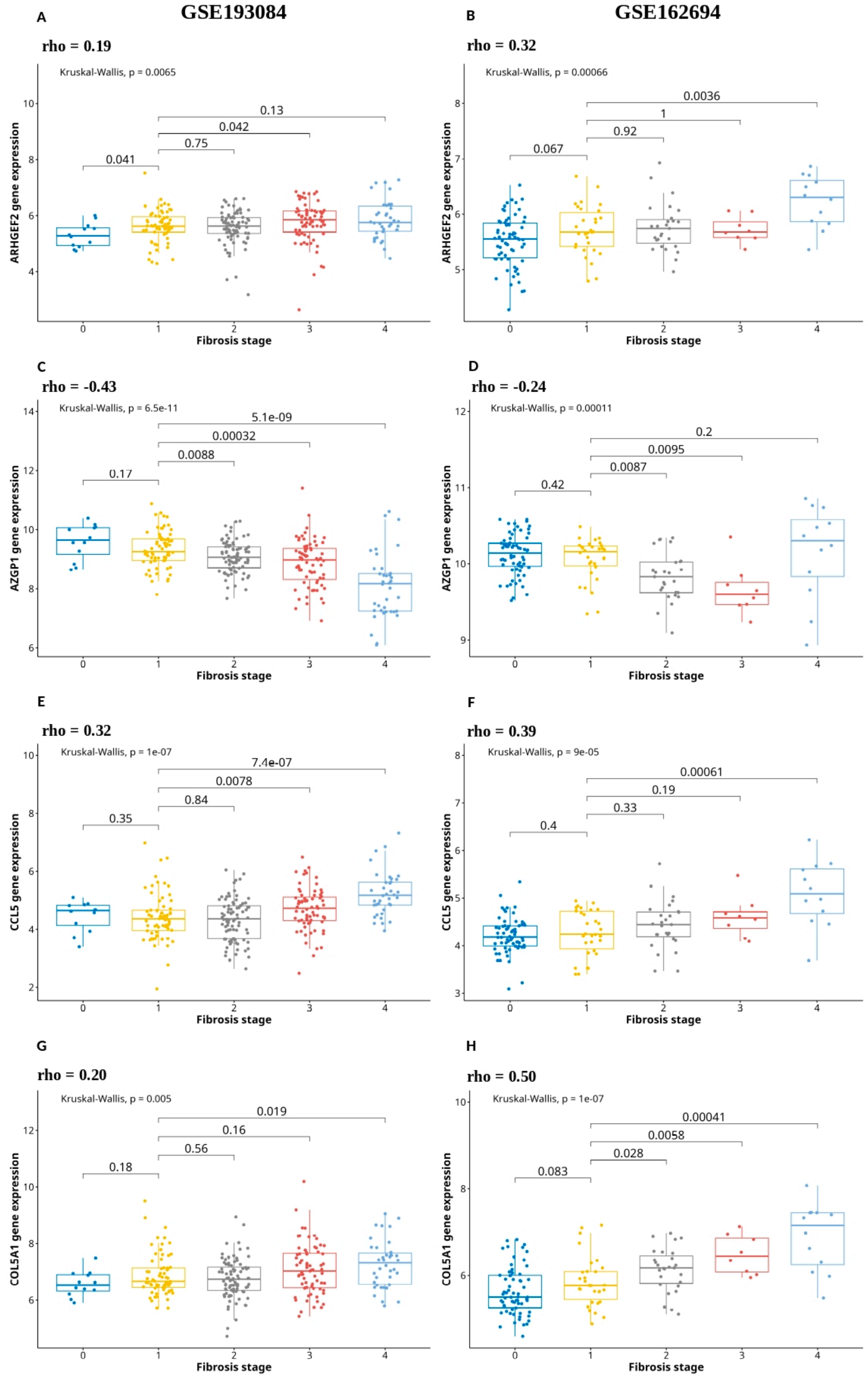

Figure S1. Box plots illustrating the association of hallmark elements of the pan-fibrotic gene-expression signature, with fibrosis progression in the RNA-seq liver validation datasets. (A-H) Box plots showing the expression of selected genes across fibrosis stages (0–4) in two liver RNA-seq validation datasets. Spearman correlation coefficients (rho) indicate the strength of association between gene expression and fibrosis progression, while p-values from Kruskal-Wallis tests denote statistical significance.

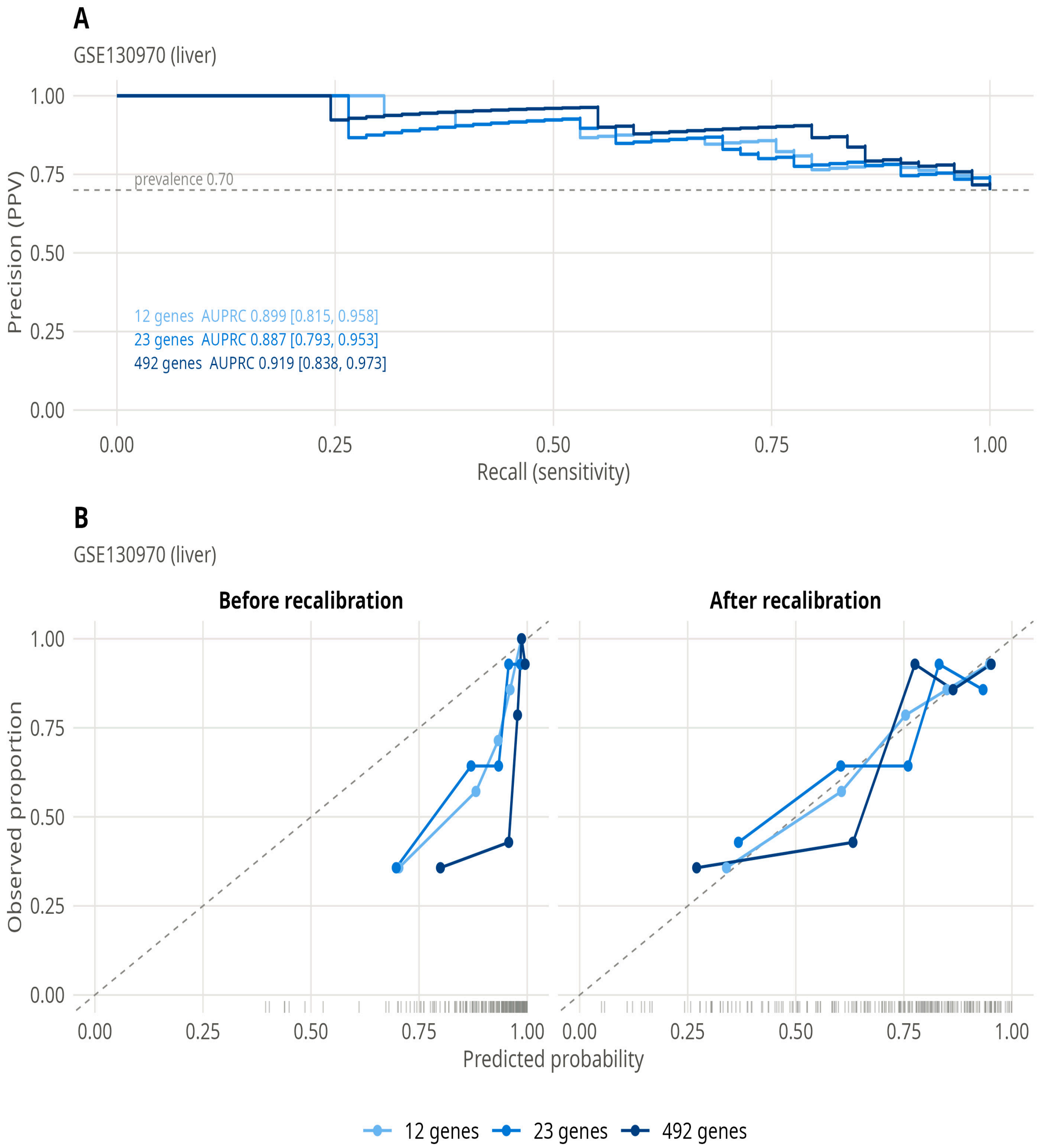

Figure S2. Precision-recall performance and calibration before and after recalibration, GSE130970. Elastic net models; color denotes gene set. (A) Precision-recall curves. The dashed line is the cohort prevalence (0.70). AUPRC with bootstrap 95% confidence intervals is given for each gene set. (B) Calibration of predicted probability against observed proportion, in five equal-count bins, before (left) and after (right) logistic recalibration of the intercept and slope (Platt scaling), with the diagonal indicating perfect calibration. Rug marks on the x-axis show the distribution of individual predicted probabilities.

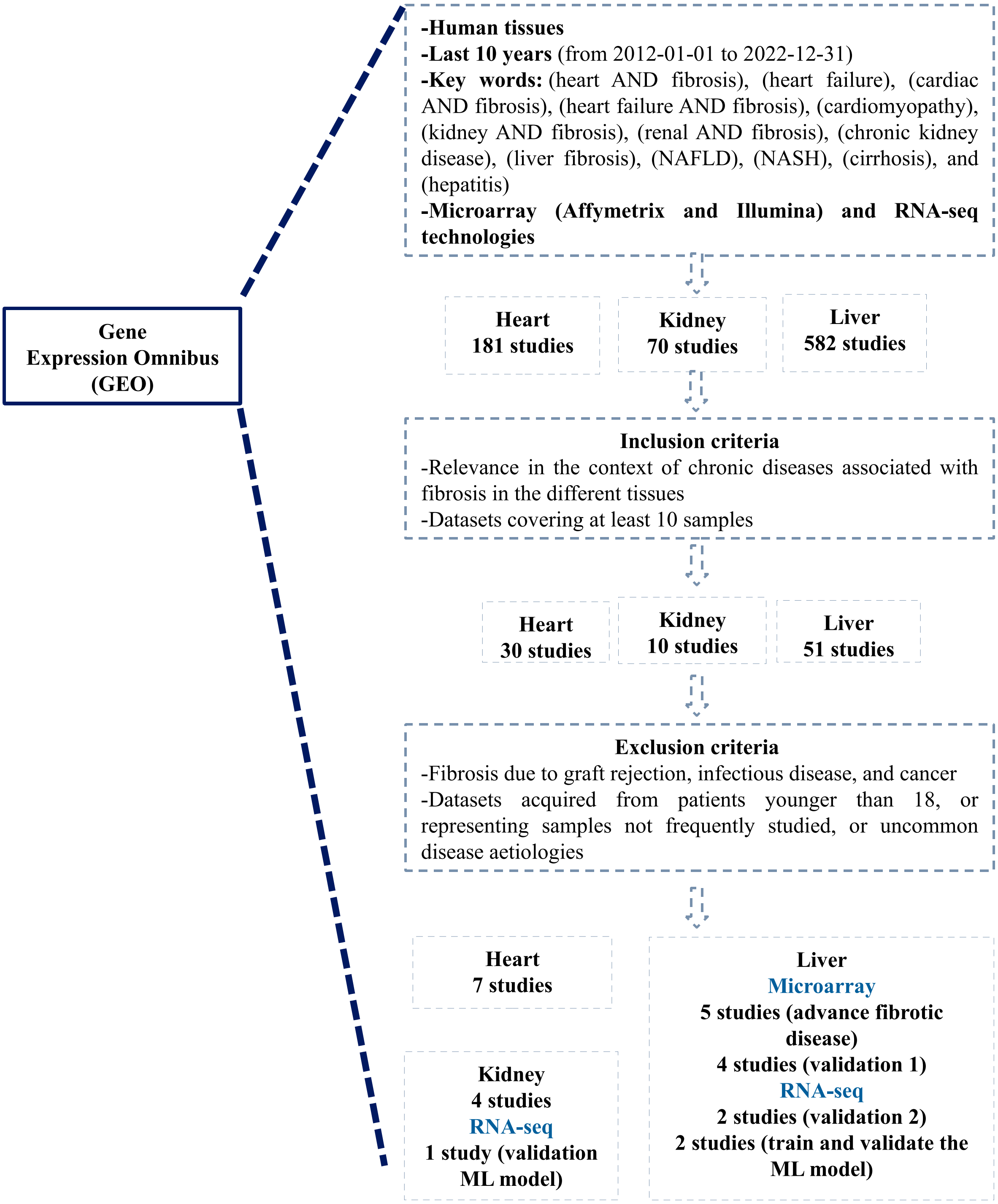

Figure S3. Overall workflow for the retrieval and curation of microarray, and RNAseq publicly available data representing fibrotic and non-fibrotic conditions.

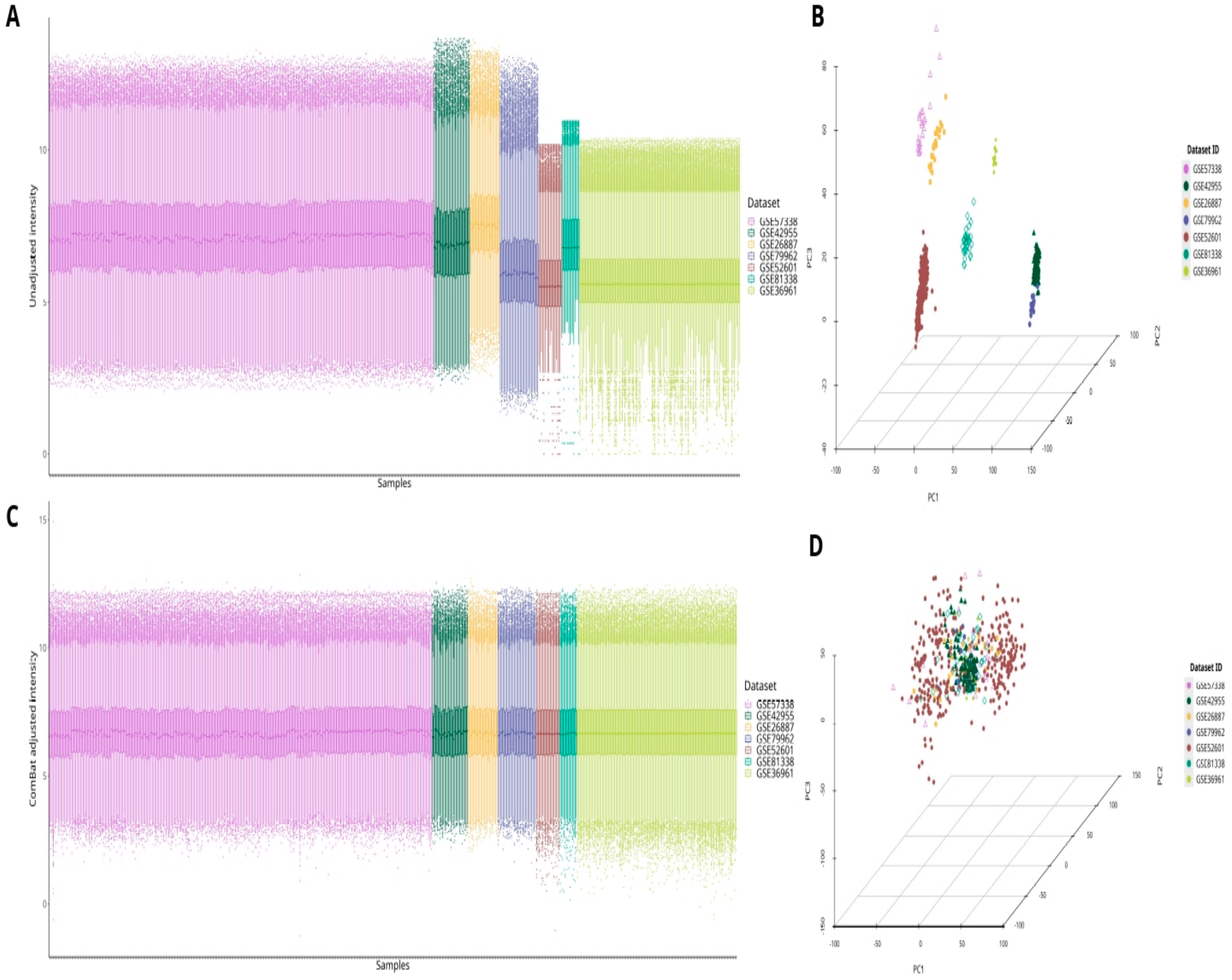

Figure S4. Batch effect correction of heart microarray datasets (discovery). (A-B) Box plots and PCA plots of unadjusted expression data, showing dataset-specific variability. (C-D) Box plots and PCA plots after ComBat adjustment, illustrating successful batch effect correction with improved sample distribution and reduced dataset-specific clustering.

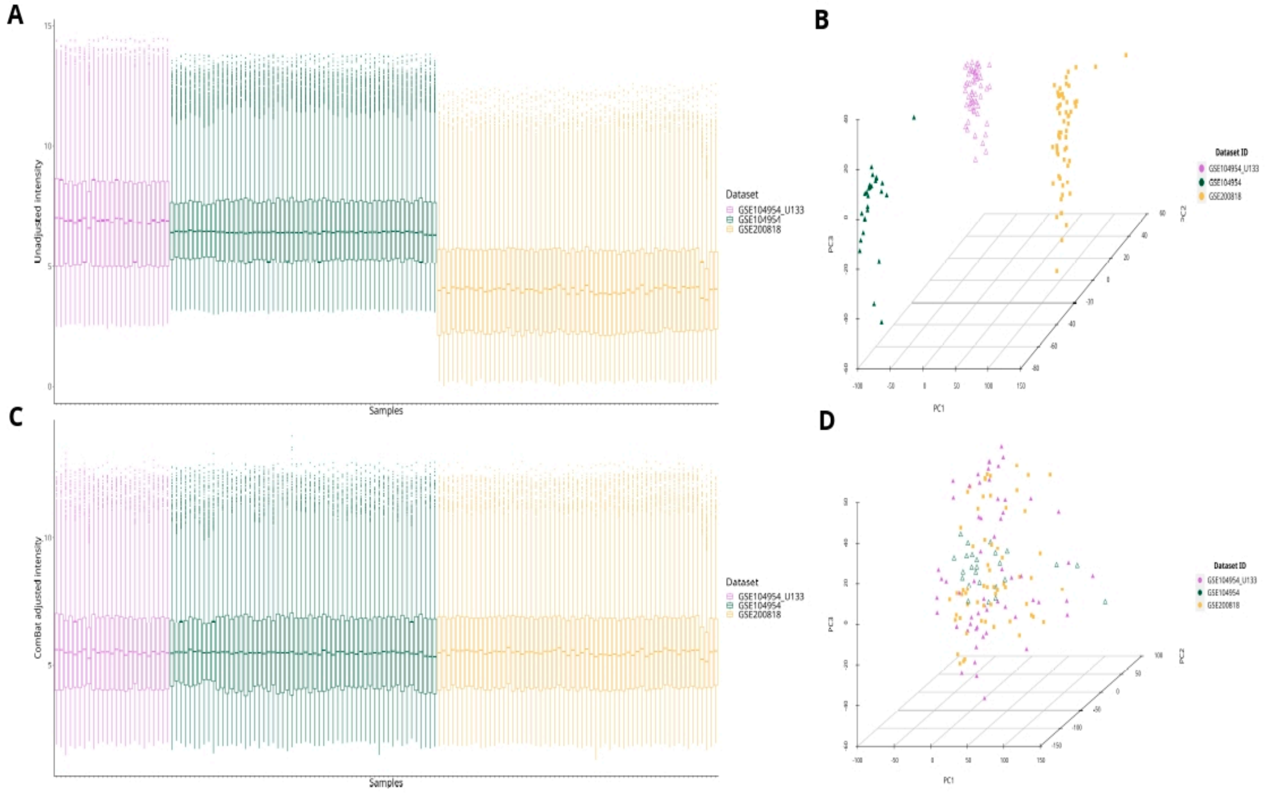

Figure S5. Batch effect correction of kidney tubuli microarray datasets (discovery). (A-B) Box plots and PCA plots of unadjusted expression data, showing dataset-specific variability. (C-D) Box plots and PCA plots after ComBat adjustment, illustrating successful batch effect correction with improved sample distribution and reduced dataset-specific clustering.

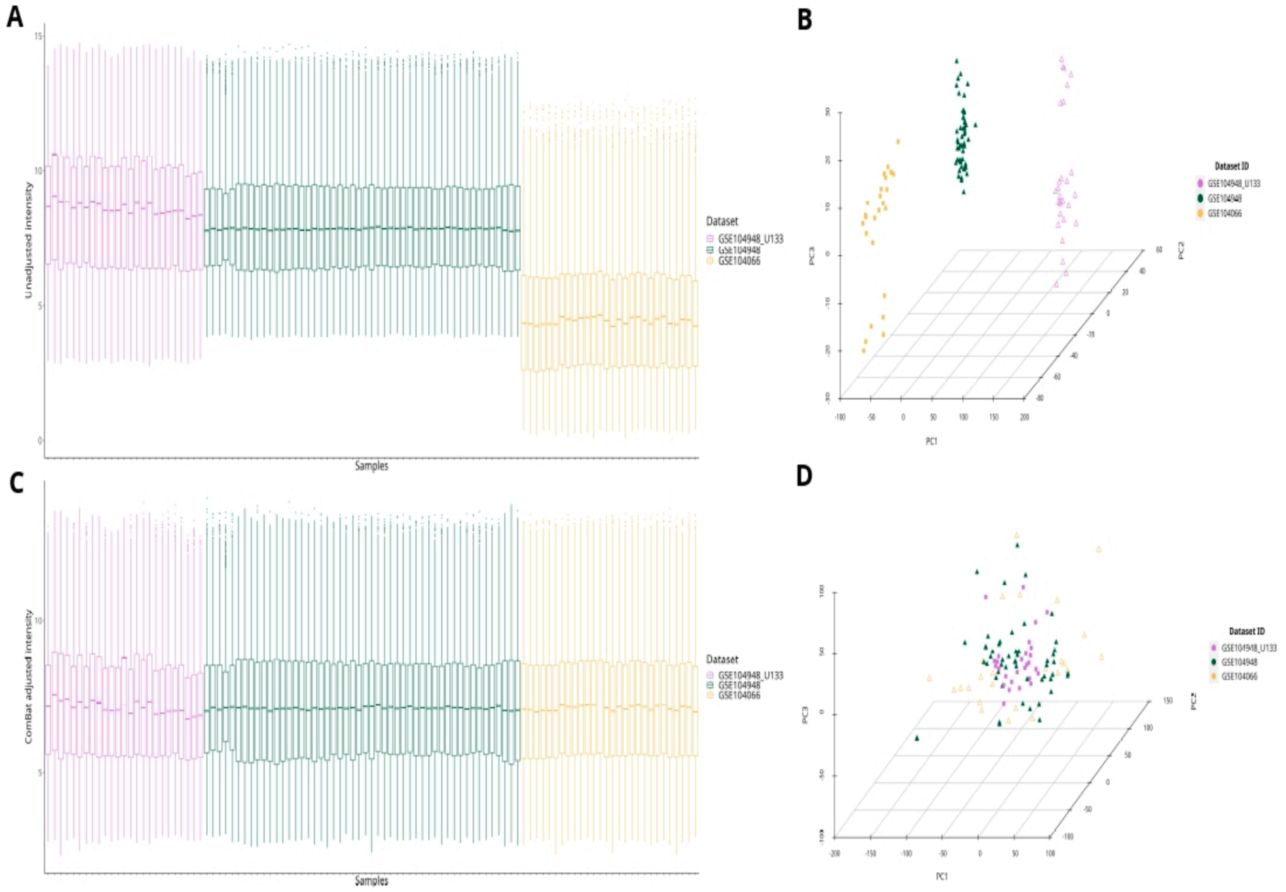

Figure S6. Batch effect correction of kidney glomeruli microarray datasets (discovery). (A-B) Box plots and PCA plots of unadjusted expression data, showing dataset-specific variability. (C-D) Box plots and PCA plots after ComBat adjustment, illustrating successful batch effect correction with improved sample distribution and reduced dataset-specific clustering.

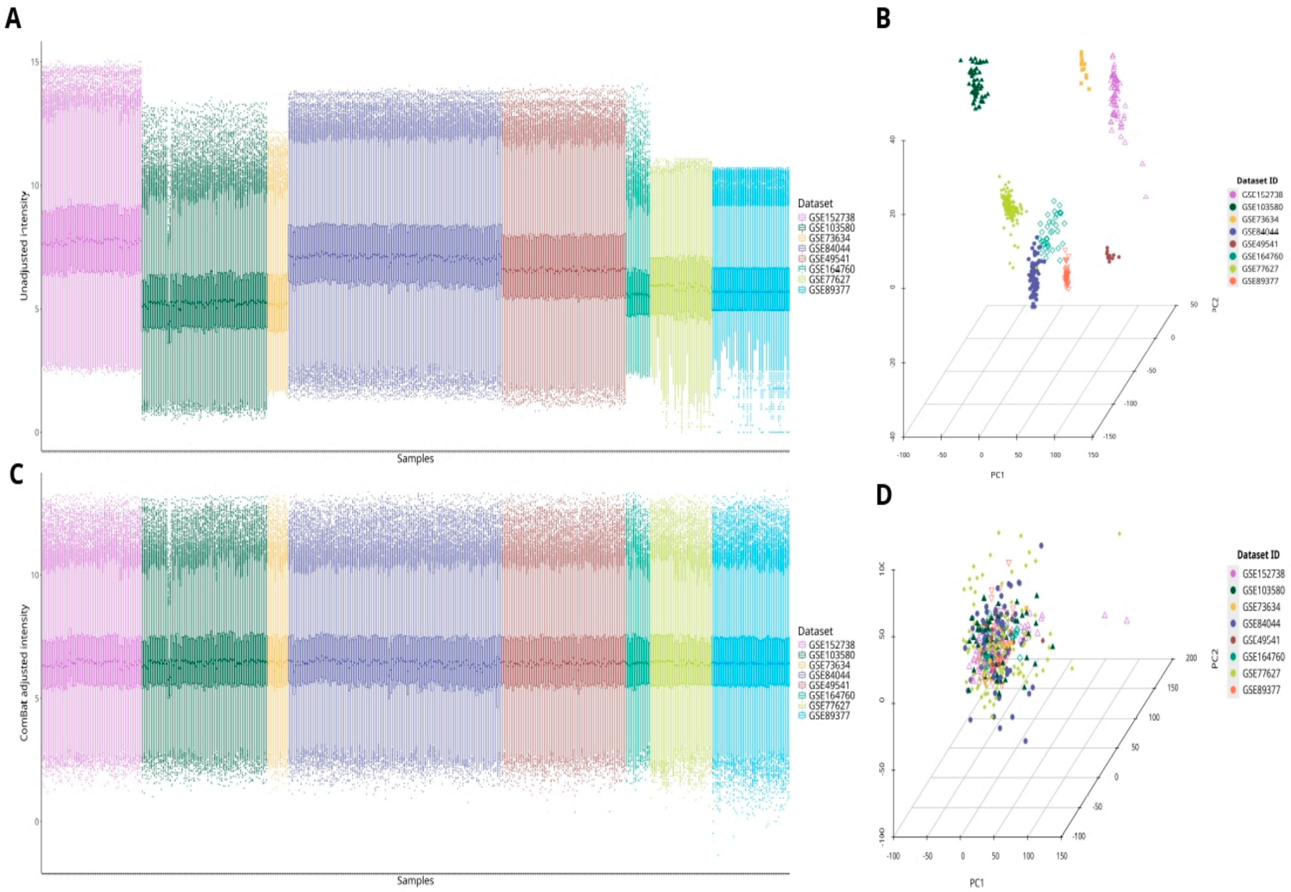

Figure S7. Batch effect correction of liver microarray datasets (discovery + initial validation cohorts). (A-B) Box plots and PCA plots of unadjusted expression data, showing dataset-specific variability. (C-D) Box plots and PCA plots after ComBat adjustment, illustrating successful batch effect correction with improved sample distribution and reduced dataset-specific clustering.

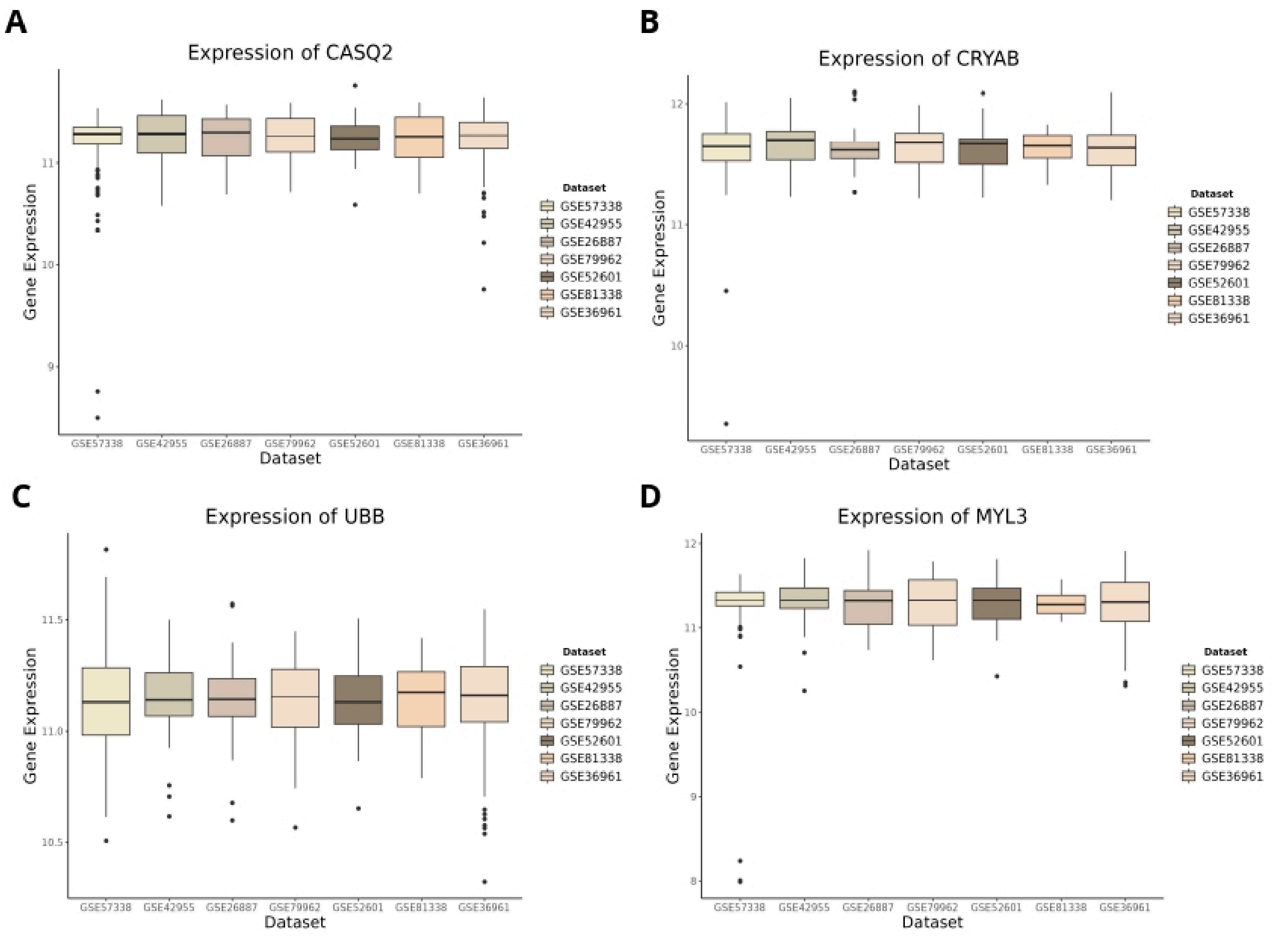

Figure S8. ComBat-adjusted expression of housekeeping genes across individual heart microarray datasets. (A-D) Box plots showing the high and consistent mean expression levels of selected housekeeping genes after batch effect correction using the ComBat algorithm.

Supplementary Tables

Gene	Heart n=555 (discovery) (Cases = 362, controls= 193)		Kidney tubuli (discovery) n=144 (Cases = 118, controls= 26)		Kidney glomeruli (discovery) n=103 (Cases = 76, controls= 27)		Liver (discovery) n=249 (Cases = 109, controls= 140)		Liver (validation) n=325 (Cases = 185, controls= 140)		Liver GSE193084 (RNA-seq validation) n=271 (Cases = 259, controls= 12)		Liver GSE162694 (RNA-seq validation) n=143 (Cases = 77, controls= 66)	
	BH	log2FC	BH	log2FC	BH	log2FC	BH	log2FC	BH	log2FC	BH	log2FC	BH	log2FC
MFAP4	1,95E-40	0,78	1,27E-02	0,45	1,82E-02	0,37	4,90E-09	0,53	3,64E-04	0,36	1,06E+00	-0,02	2,79E-03	0,45
MT1X	4,62E-34	-0,88	5,46E-03	-0,46	2,00E-02	-0,35	6,85E-06	-0,54	8,50E-04	-0,36	3,00E-02	-0,65	4,21E-03	-0,4
PLA2G4C	2,20E-30	0,41	8,73E-03	0,47	4,62E-02	0,29	6,65E-03	0,29	3,63E-02	0,25	1,17E-01	0,26	5,42E-04	0,41
ELL2	4,28E-27	-0,55	2,63E-05	-0,66	1,17E-03	-0,41	1,73E-04	-0,34	8,11E-03	-0,21	1,94E-01	-0,17	5,67E-01	-0,04
MOXD1	3,09E-24	0,58	8,07E-04	0,91	1,24E-03	0,96	4,90E-09	0,68	1,81E-03	0,47	1,74E-02	0,46	2,61E-09	1,39
CCL5	1,19E-21	0,43	7,78E-04	0,74	4,92E-05	0,85	9,24E-05	0,39	2,71E-03	0,3	1,99E-01	0,2	2,65E-05	0,61
SYT11	2,79E-20	0,28	1,09E-03	0,53	1,36E-06	0,84	4,56E-05	0,26	3,69E-02	0,16	1,03E+00	0,01	2,69E-03	0,28
AEBP1	1,82E-17	0,52	1,48E-02	0,34	3,19E-04	0,7	3,57E-07	0,59	5,96E-05	0,46	2,04E-01	0,16	1,69E-04	0,69
KLF15	4,02E-16	-0,39	4,72E-02	-0,22	2,60E-03	-0,3	2,92E-03	-0,24	6,44E-03	-0,21	3,01E-02	-0,35	1,32E-01	-0,12
VEGFC	1,78E-14	0,26	6,38E-03	0,33	2,33E-05	0,88	4,26E-03	0,33	3,87E-02	0,23	3,20E-01	0,15	2,68E-04	0,36
AZGP1	1,50E-12	-0,4	4,97E-02	-0,51	4,09E-03	-0,77	6,12E-03	-0,21	1,21E-02	-0,17	1,12E-03	-0,71	6,89E-05	-0,27
SERPINE 2	4,47E-12	0,51	2,36E-03	0,51	2,57E-02	0,63	3,46E-04	0,33	2,14E-03	0,29	1,07E-01	1	8,11E-05	0,39
SOX4	2,47E-08	0,14	3,20E-04	0,84	3,61E-07	1,11	1,38E-02	0,31	7,12E-03	0,35	1,06E+00	-0,01	1,41E-10	0,86
HCP5	5,43E-08	0,21	1,47E-03	0,54	1,82E-02	0,34	2,75E-07	0,38	7,51E-04	0,26	5,22E-03	0,94	8,48E-05	0,3
DUSP1	7,67E-07	-0,27	1,24E-09	-1,44	1,06E-03	-0,62	2,72E-02	-0,23	1,78E-02	-0,26	3,63E-01	-0,14	3,99E-06	1,01
ALDH1L1	2,02E-05	-0,14	2,61E-02	-0,37	3,29E-02	-0,41	1,26E-03	-0,35	1,24E-02	-0,27	4,22E-04	-0,85	7,48E-02	-0,15
COL5A1	2,03E-05	0,11	3,15E-02	0,17	2,52E-05	0,64	5,17E-03	0,31	9,87E-03	0,28	2,01E-02	0,51	1,88E-06	0,72

ARHGEF 2	2,02E-04	0,08	2,68E-03	0,25	1,57E-05	0,48	1,01E-02	0,15	1,81E-02	0,13	4,68E-04	0,56	1,01E-03	0,28
S100A13	2,59E-04	0,07	1,88E-03	0,41	2,66E-02	0,22	1,26E-03	0,2	1,66E-02	0,16	1,04E+00	0	NA	0,09
THBS2	1,12E-03	0,17	3,37E-04	0,79	2,21E-04	0,86	2,68E-05	0,58	1,74E-03	0,49	4,49E-02	0,33	1,17E-08	1,14
CA2	2,46E-03	-0,09	6,02E-03	-0,28	3,41E-03	-0,6	6,73E-04	-0,24	1,80E-02	-0,15	5,00E-04	-0,85	7,26E-02	-0,12
CRIP1	6,12E-03	0,1	3,66E-04	0,76	8,33E-06	0,94	7,55E-03	0,26	1,35E-02	0,25	NA	NA	NA	NA
MT1E	1,86E-02	-0,17	3,37E-04	-0,72	1,17E-02	-0,42	6,40E-03	-0,23	8,23E-03	-0,22	1,10E-02	-0,9	2,95E-03	-0,4

Table S6. List of 23 validated hallmark molecular features differentially expressed between fibrotic and non-fibrotic samples, identified through integrated analysis of microarray and RNA-seq datasets.

	GSE193084 (stage 0 = 12, stage 1 = 66, stage 2 = 84, stage 3 = 71, stage 4 = 38)			GSE162694 (stage 0 = 66, stage 1 = 30, stage 2 = 27, stage 3 = 8, stage 4 = 12)		
**Gene**	**p-values**	**BH**	**rho**	**p-values**	**BH**	**rho**
MT1E	1,02E-09	4,84E-09	-0,36	2,04E-03	2,77E-03	-0,26
ARHGEF2	1,71E-03	2,03E-03	0,19	8,50E-05	1,35E-04	0,32
HCP5	2,97E-06	7,05E-06	0,29	1,78E-04	2,61E-04	0,31
AZGP1	2,83E-13	2,69E-12	-0,42	4,70E-03	5,95E-03	-0,24
MOXD1	1,41E-10	8,93E-10	0,39	3,58E-10	2,27E-09	0,49
MT1X	1,65E-09	5,99E-09	-0,36	2,48E-02	2,94E-02	-0,19
COL5A1	9,96E-04	1,26E-03	0,2	2,44E-10	2,27E-09	0,5
THBS2	2,47E-05	4,27E-05	0,25	2,60E-09	9,87E-09	0,47
KLF15	1,83E-04	2,67E-04	-0,23	1,92E-01	2,02E-01	-0,11
ALDH1L1	8,17E-15	1,55E-13	-0,45	4,96E-02	5,54E-02	-0,16
CA2	1,89E-09	5,99E-09	-0,35	3,98E-01	3,98E-01	-0,07
SYT11	2,52E-02	2,66E-02	0,14	2,32E-05	4,41E-05	0,35
PLA2G4C	1,60E-05	3,04E-05	0,26	9,99E-07	2,71E-06	0,4
VEGFC	2,84E-04	3,85E-04	0,22	7,35E-06	1,55E-05	0,37
AEBP1	8,97E-05	1,42E-04	0,24	1,57E-09	7,46E-09	0,48
SERPINE2	5,97E-02	5,97E-02	0,11	5,05E-09	1,60E-08	0,46
CCL5	1,18E-07	3,21E-07	0,31	1,34E-06	3,19E-06	0,39
MFAP4	1,11E-05	2,35E-05	0,27	5,93E-05	1,02E-04	0,33
SOX4	2,43E-03	2,72E-03	0,19	1,71E-10	2,27E-09	0,5

Table S7. Correlation of 19 validated hallmark molecular features with the degree of fibrosis in the bulk RNA-seq datasets.

Dataset accession	Assay technology	Original number of samples	Excluded	Exclusion criteria	Sample type	Final number of samples	Cases	Disease/condition	Controls	Control samples	Reference (Pubmed ID)
**GSE57338**	Affymetrix Human Gene 1.1 ST Array	313	4	Age below 18yrs	Left ventricular tissue	309	176	Idiopathic dilated cardiomiopathy, ischemic cardiomiopathy	133	Unused heart donors	PMID: 25528681
**GSE52601**	Illumina HumanHT-12 V4.0	24	5	Age below 18yrs	Left ventricular tissue	19	16	Ischemic cardiomiopathy, dilated cardiomiopathy	3	Non-failing heart donors	PMID: 25012294
**GSE42955**	Affymetrix Human Gene 1.0 ST Array	29	0	-	Left ventricular tissue	29	24	Ischemic cardiomiopathy, dilated cardiomiopathy	5	Unused heart donors	PMID: 24339868
**GSE26887**	Affymetrix Human Gene 1.0 ST Array	24	0	-	Left ventricular tissue	24	19	Diabetic and non-diabetic heart failure	5	Non-failing heart donors	PMID: 22427379
**GSE79962**	Affymetrix Human Gene 1.0 ST Array	51	20	Sepsis	Left ventricular tissue	31	20	Ischemic heart disease, dilated cardiomyopathy	11	Non-failing heart donors	PMID: 28067713
**GSE81338**	Illumina HumanHT-12 V4.0	30	16	Samples from right ventricle and replicates from the same patient in left ventricle	Left ventricular tissue	14	14	Dilated cardiomyopathy	-	-	PMID: 27417303, PMID: 27699191
**GSE36961**	Illumina HumanHT-12 V3.0	145	16	Age below 18yrs	Left ventricular tissue	129	93	Hypertrophic Cardiomyopathy	36	Unused heart donors	NA

Table S14. Selected heart tissue microarray datasets.

Dataset accession	Assay technology	Original number of samples	Excluded	Exclusion criteria	Sample type	Final number of samples	Cases	Disease/condition	Controls	Control samples	Reference (Pubmed ID)
**GSE104954**	Affymetrix Human Genome U133 Plus 2.0	195	112	Tumor nephrectomy, thin membrane disease, systemic lupus erythematosus, focal segmental glomerulosclerosis, minimal change disease, ANCA-associated vasculitis,	Microdissection on kidney biopsies (tubular compartment)	83	62	Chronic kidney disease (Diabetic nephropathy, hypertensive nephropathy, IgA nephropathy).	21	Living donor biopsies	PMID: 29724730
**GSE200818**	Affymetrix Human Gene 2.1 ST	193	132	Focal and Segmental Glomerulosclerosis, Minimal Change Disease	Microdissection on kidney biopsies (tubular compartment)	61	56	Chronic kidney disease (Glomerular Disease (IgA nephropathy)).	5	Living donor biopsies	PMID: 23393107 and PMID: 23325076; PMID: 28339906
**GSE104948**	Affymetrix Human Genome U133 Plus 2.0	196	121	Tumor nephrectomy, thin membrane disease, systemic lupus erythematosus	Microdissection on kidney biopsies (glumeruli compartment)	75	54	Chronic kidney disease (Diabetic nephropathy, hypertensive nephropathy, IgA nephropathy).	21	Living donor biopsies	PMID: 29724730
**GSE104066**	Affymetrix Human Gene 2.1 ST	76	48	Membranous Nephropathy, Focal and Segmental Glomerulosclerosis, Minimal Change Disease	Microdissection on kidney biopsies (glumeruli compartment)	28	22	Chronic kidney disease (Glomerular Disease (IgA nephropathy)).	6	Living donor biopsies	PMID: 30301568

Table S15. Selected kidney tissue microarray datasets from tubuli and glomeruli.

Dataset accession	Assay technology	Original number of samples	Excluded	Exclusion criteria	Final number of samples	Sample type	Cases	Disease/ condition	Control ¥	Control samples	Reference (Pubmed ID)

**GSE152738**	Affymetrix Human Genome U133 Plus 2.0	58	-	-	58	Liver specimen	-	-	58	Healthy liver donors	PMID: 33888584
**GSE77627**	Illumina HumanHT-12 WG-DASL V4.0 R2	54	18	Idiopathic noncirrhotic portal hypertension	36	Liver specimen	22	Cirrhosis	14	Histologically normal livers	PMID: 34052252
**GSE103580**	Affymetrix Human Genome U219	86	13	NAFLD and NASH without information on fibrosis stage	73	Tissue biopsies	67	Cirrhosis	6	Histologically normal livers	PMID: 29158192
**GSE89377**	Illumina HumanHT-12 V3.0	107	62 (+20*)	HCC, dysplastic nodules (*including those used for validation)	25	Liver specimen	12	Cirrhosis	13	Healthy liver donors	PMID: 29059470; PMID: 35205612
**GSE164760**	Affymetrix Human Genome U219	170	156	Adjacent non-tumor NASH, HCC and NASH without information on fibrosis stage	14	Liver specimen	8	Cirrhosis	6	Healthy liver donors	PMID: 33992698

¥The same set of controls (GSE152738, GSE77627, GSE103580, GSE89377, GSE164760, GSE84044) were used for the microarray discovery and validation cohorts.

Table S16. Selected liver tissue microarray datasets.

**Dataset accession**	**Assay technology**	**Original number of samples**	**Excluded**	**Exclusion criteria**	**Sample type**	**Final number of samples**	**Cases**	**Disease/ Condition**	**Control¥**	**Control sample s**	**Reference (Pubmed ID)**
GSE89377	Illumina HumanHT-12 V3.0	107	62 (+12*)	HCC and dysplastic nodules (*including those used	Liver specimen	33	20	Chronic hepatitis	13	Healthy liver donors	PMID: 29059470; PMID: 35205612

				for discovery)							
GSE73634	Affymetrix Human Gene 2.0 ST	48	36	Post-treatment with simtuzumab	Tissue biopsies	12	12	NASH	0	-	PMID: 27232579
GSE84044	Affymetrix Human Genome U133 Plus 2.0	124			Tissue biopsies	124	81	Chronic hepatitis	43	NAFLD fibrosis stage 0	PMID: 28262670
GSE49541	Affymetrix Human Genome U133 Plus 2.0	72			Tissue biopsies	72	72	NAFLD	0	-	PMID: 23913408 and PMID: 23916847

¥The same set of controls (GSE152738, GSE77627, GSE103580, GSE89377, GSE164760, GSE84044) were used for the microarray discovery and initial validation cohorts.

Table S17. Selected microarray initial validation cohort datasets.

**Dataset accession**	**Assay technology**	**Original number of samples**	**Excluded**	**Exclusion criteria**	**Sample type**	**Final number of samples**	**Cases**	**Disease/condition**				**Controls**	**Control samples**	**Reference (Pubmed ID)**
**GSE193084**	Illumina NextSeq 500	271	0	-	Tissue biopsies	271	259	NASH				12	Histologically normal livers and NAFLD fibrosis stage 0	PMID: 29724730
								**Stage 1**	**Stage 2**	**Stage 3**	**Stage 4**			
								66	84	71	38			
**GSE162694**	Illumina HiSeq 3000	143	0	-	Tissue biopsies	143	77	NASH				66	NAFLD fibrosis stage 0	PMID: 34508113
								**Stage 1**	**Stage 2**	**Stage 3**	**Stage 4**			
								30	27	8	12			

Table S18. Independent RNA-seq second validation cohort.

Dataset accession	Assay technology	Original number of samples	Excluded	Exclusion criteria	Sample type	Final number of samples	Cases	Disease/condition	Stage 1	Stage 2	Stage 3	Stage 4	Controls	Control samples	Reference (Pubmed ID)
**GSE185477**	Illumina HiSeq 2500	4	1	Low number of cells	Liver specimen	3	0	-	-	-	-	-	3	Neurologically deceased donor liver acceptable for liver transplantation.	PMID: 34792289
**GSE244832**	Illumina NextSeq 500	18	-	-	Liver specimen	18	9	NAFLD, NASH, cirrhosis	-	1	4	4	9	Histologically normal livers and NAFLD fibrosis stage 0	PMID: 39522884
**GSE210077**	Illumina NextSeq 500	35	29	No description of fibrosis stage	Liver specimen	6	3	No specified	-	1	1	1	3	Healthy liver donors	PMID: 39747812
**GSE202379**	Illumina NovaSeq 6000	47	5	End stage	Liver specimen and tissue biopsies	42	37		9	12	12	4	5	Histologically normal livers and NAFLD fibrosis stage 0	PMID: 38778114

Table S19. Independent snRNA-seq validation cohort.

Ishak score	Scheuer system and METAVIR system	Pathology Committee of the NASH Clinical Research Network	Not specified	Unified system
0 (no fibrosis)	F0 (no fibrosis-“mild“)	Stage 0 (no fibrosis)		No fibrosis
1 (mild fibrosis)	F1 (mild to moderate fibrosis)	Stage 1 (mild fibrosis)	low-grade fibrosis of chronic hepatitis	Low fibrosis
2(mild to moderate fibrosis)	Either or between F1 and F2	Either or between stage 1 and 2 (mild to moderate fibrosis)		Moderate fibrosis
3 (moderate fibrosis)	F2 (moderate fibrosis9	Stage 2 (moderate fibrosis)		Moderate fibrosis
4 (moderate to severe fibrosis)	F3 (severe fibrosis)	Stage 3 (severe fibrosis)	high-grade fibrosis of chronic hepatitis	Severe fibrosis
5 (incomplete cirrhosis)	Either or between F3 or F4 (severe fibrosis or cirrhosis)	Either or between stage 3 or 4 (severe fibrosis or cirrhosis)		Severe fibrosis
6 (cirrhosis)	F4 (cirrhosis)	Stage 4 (cirrhosis)		Severe fibrosis

Table S20. Unification of the different systems describing the degree of liver fibrosis in the microarray validation cohort.

Dataset accession	Assay technology	Original number of samples	Excluded	Exclusion criteria	Sample type	Final number of samples	Cases	Disease/condition				Controls	Control samples	Reference (Pubmed ID)
**GSE135251**	Illumina NextSeq 500	216	7	Successfully assigned alignment rate below 55%	Tissue biopsies	209	164	NAFLD				45	Histologically normal livers and NAFLD fibrosis stage 0	PMID: 33268509
								**Stage 1**	**Stage 2**	**Stage 3**	**Stage 4**			
								46	51	53	14			
**GSE130970**	Illumina HiSeq 2500	78	8	Successfully assigned alignment rate below 55%	Tissue biopsies	70	49	NAFLD				21	NAFLD fibrosis stage 0	PMID: 31467298
								**Stage 1**	**Stage 2**	**Stage 3**	**Stage 4**			
								25	9	13	2			
**GSE137570**	Illumina HiSeq 2000	24	-	-	Tissue biopsies	24	21	CKD				3	%IFTA <5%	PMID: 36550108
								**Stage 1**	**Stage 2**	**Stage 3**	**Stage 4**			
								5	5	-	11			

Table S21. Independent training and validation cohorts for machine learning using RNA-seq.

Cell type	Canonical markers
Hepatocytes	AL391117.1, ADRA1A, ACSM2B, HNF4A, CYP2A6, ABCC2, ASGR1
Monocytes	FCGR3A, MS4A7,CD163, CSF1R
T cells	IL7R, CD96, CD247
B cells	MS4A1
Cholangiocytes	CTNND2, DCDC2, CFTR, SLC12A2
Endothelial	ST6GALNAC3, PTPRB, PECAM1, FLT1, EGFL7, ERG
Stellate cells	PRKG1, LAMA2, ARHGAP10, ADAMTS9-AS2, PDGFRA, ADAMTSL2

Table S22. Canonical markers for cell cluster annotation, related to the STAR methods.
